# Comprehensive characterization of lncRNA-mRNA related ceRNA network across 12 major cancers

**DOI:** 10.18632/oncotarget.11637

**Published:** 2016-08-26

**Authors:** Yunpeng Zhang, Yanjun Xu, Li Feng, Feng Li, Zeguo Sun, Tan Wu, Xinrui Shi, Jing Li, Xia Li

**Affiliations:** ^1^ College of Bioinformatics Science and Technology, Harbin Medical University, Harbin 150081, China; ^2^ Department of Ultrasonic Medicine, The 1^st^ Affiliated Hospital of Heilongjiang University of Chinese Medicine, Harbin 150040, China

**Keywords:** lncRNA, ceRNA network, pan-cancer

## Abstract

Recent studies indicate that long noncoding RNAs (lncRNAs) can act as competing endogenous RNAs (ceRNAs) to indirectly regulate mRNAs through shared microRNAs, which represents a novel layer of RNA crosstalk and plays critical roles in the development of tumor. However, the global regulation landscape and characterization of these lncRNA related ceRNA crosstalk in cancers is still largely unknown. Here, we systematically characterized the lncRNA related ceRNA interactions across 12 major cancers and the normal physiological states by integrating multidimensional molecule profiles of more than 5000 samples. Our study suggest the large difference of ceRNA regulation between normal and tumor states and the higher similarity across similar tissue origin of tumors. The ceRNA related molecules have more conserved features in tumor networks and they play critical roles in both the normal and tumorigenesis processes. Besides, lncRNAs in the pan-cancer ceRNA network may be potential biomarkers of tumor. By exploring hub lncRNAs, we found that these conserved key lncRNAs dominate variable tumor hallmark processes across pan-cancers. Network dynamic analysis highlights the critical roles of ceRNA regulation in tumorigenesis. By analyzing conserved ceRNA interactions, we found that miRNA mediate ceRNA regulation showed different patterns across pan-cancer; while analyzing the cancer specific ceRNA interactions reveal that lncRNAs synergistically regulated tumor driver genes of cancer hallmarks. Finally, we found that ceRNA modules have the potential to predict patient survival. Overall, our study systematically dissected the lncRNA related ceRNA networks in pan-cancer that shed new light on understanding the molecular mechanism of tumorigenesis.

## INTRODUCTION

Cancer is a complex disease, the initiation and progression of which is closely related with not only the aberrant level of protein coding mRNAs, but also related with the non-coding RNAs. The non-coding RNAs can generally be classified as two classes based on their size and compose up to ~98% of the human genome, which indicate their vital roles in the normal physiology and disease progression. MiRNAs are a class of small non-coding RNA that are important regulators of gene expression by repressing translation or destabilizing the mRNAs at the post transcriptional level. A large amount of studies have proved that miRNAs play critical roles in tumor and the function of miRNAs were relative well understood. Long noncoding RNAs (lncRNAs) is a class of novelty discovered non-coding RNA which have been demonstrated that participated in multiple biological processes and closely related with diseases. However, the functions of a large amount of lncRNAs and their functional roles in cancer is still unclear.

Recently, Salmena et al. firstly proposed the ceRNA hypothesis that mRNA and lncRNA can talk with each other through microRNA response elements [1]. And then increasing studies also demonstrated that lncRNAs contain miRNA-response elements and can compete miRNAs with mRNAs, which act as compete endogenous RNAs (ceRNAs) and thus implicated in multiple biological processes and tumorigenesis. For example, lncRNA HULC competitively regulated PRKACB by sharing common binding site of miR-372 and thus induced phosphorylation of CREB in liver cancer [2]. The well-known lncRNA H19, which play important roles in multiple cancers such as liver, breast and colorectal cancers [3], has recently been proved to act as miRNA sponge for miR-138 and miR-200a to promote the transition from epithelial to mesenchymal in colorectal cancer [4]. The competitively regulated function of lncRNA is not only active in tumor, but also occurs in other different biological contexts. Two such example lncRNAs are linc-MD1 and linc-ROR. Linc-MD1, a specifically expressed lncRNA in muscle, has been demonstrated that its ceRNA activity plays important role in regulating muscle differentiation [5]. In addition, Wang and colleagues proved that linc-ROR could competing miRNAs with OCT4, SOX2 and NANOG which are essential transcription factors in self-renewal of pluripotent embryonic stem cell [6]. The above described example indicate that the ceRNA regulation may represent a widespread layer of gene regulation which is not only important for normal physiological states, but is also crucially relevant with pathogenesis such as cancer. Thus, systematically analyzing the lncRNA related ceRNA network of disease may provide valuable insight into the function of lncRNAs and the molecular mechanism of diseases.

Currently, several data sources were developed that aims to provide potential miRNA-lncRNA interactions. For example, miRcode identified miRNA target sites on lncRNAs based on targetscan prediction algorithm [7], DIANA-LncBase [8] and starBase [9] integrated Ago CLIP-supported data to identify miRNA-lncRNA interactions, while Wang et al. [10] provided a computational framework to identify lncRNA-associated competing triplets. In addition, The Cancer Genome Atlas (TCGA) research group developed a comprehensive resource that stored multidimensional molecular profiles of a large amount tumor samples. These datasets were all valuable resource and provided the possibility for integrating analysis of ceRNA network in cancer. Sumazin and colleagues systematically investigated the mRNA related ceRNA network in glioblastoma and suggest that these ceRNA regulation may mediate the crosstalk between oncogenic pathways [11]. Paci and colleagues developed a computational pipeline to predict the ceRNA interactions between long non-coding RNAs and messenger RNAs in human breast cancer [12], while Shao et al. aims to identify dysregulated ceRNA-ceRNA interaction in lung cancer and suggested several ceRNA interaction modules may have the potential to serve as diagnostic biomarkers [13]. Exploring the lncRNA related ceRNA network of cancer will undoubtedly lead to important insight into tumorigenesis and lncRNA function. However, our knowledge about the ceRNA function of lncRNA in cancer is still limited. Most of these studies only focused on analyzing one particular tumor, and thus lack the global understanding of lncRNA related competitive activity across different cancers. Furthermore, miRNA and ceRNA especially lncRNA were known to have specific expression patterns in different tissues and disease states. This indicate that ceRNAs may exhibit different activity and regulated patterns across different cancers, and thus a pan-cancer analysis of the ceRNA crosstalk is essential. Moreover, the dynamic range of ceRNAs between various tumorigenesis and normal physiology is still an open question to be address.

Here, we systematically integrated multidimensional expression profile of more than 5000 samples across 12 cancers to investigate the lncRNA related ceRNA crosstalk networks in both tumor and normal physiological states. By comprehensively analyzing these ceRNA crosstalk, we revealed many important properties and ceRNA regulation patterns in human cancer. The ceRNA regulation varied greatly from normal to tumor states. Comparison across cancers found that ceRNA regulations show higher similarity in cancer types with similar tissue origin, while lncRNAs tend to be shared by multiple cancer types. Pan-cancer ceRNAs were mainly comprised by molecules that play critical roles in physiological conditions or tumor biology. Expression analysis indicate that lncRNAs in the ceRNA network have the potential to be cancer biomarkers. Network hub analysis suggest that conserved hub lncRNAs may dominate different cancer hallmarks across various tumors. Network dynamic analysis found that a large proportion of changes in ceRNA regulation were observed between tumor and corresponding normal conditions. We also found variable miRNA-mediated ceRNA regulation pattern in pan-cancer. Finally, network module analysis indicate that ceRNA crosstalk may have the potential for prediction of cancer prognosis. In summary, our systematically pan-cancer ceRNA crosstalk analysis not only shed new light on the molecular mechanism of tumorigenesis, but also help to tumor prognosis stratification and discovery of therapeutic targets.

## RESULTS

### Global landscape and comparison of ceRNA networks across 12 cancers

To investigate the role of ceRNAs and the competitive pattern of lncRNAs in the tumorigenesis, we constructed lncRNA related ceRNA networks for 12 cancers and the corresponding normal states by applying a two steps pipeline. First, for each lncRNA-miRNA-mRNA interaction, of which the lncRNA and mRNA shared common miRNA binding site, we evaluated the miRNA mediated strength of the lncRNA-mRNA ceRNA pair by calculating the sensitivity correlation of Paci et al. [12]. The distribution and the cut-off values corresponding to top 5% of sensitivity correlation for each ceRNA network were shown in Supplementary Figure S2 and Supplementary Table S2. Then, we filter the lncRNA-mRNA interactions of each specific cancer and the normal state by considering the positively correlated expression. The distribution and the cut-off values corresponding to top 5% of Pearson correlation for each ceRNA network were shown in Supplementary Figure S3 and Supplementary Table S3. We found that, most cut-offs of the Pearson correlation coefficients were greater than 0.4 in these 24 ceRNA networks. Specifically, all cut-off values of the Pearson correlation coefficients in these normal ceRNA networks were greater than 0.5. Furthermore, it is worth to note that all of these top 5% Pearson correlation were statistical significance (P-value <0.01). And the corrected P-values (FDR) were also shown in Supplementary Table S3. Significance P-values were corrected by Benjamini-Hochberg method. Finally, we totally identified 7067 lncRNA-mRNA competitive interactions including 252 lncRNAs and 1176 mRNAs in 12 cancers and normal physiological states and then assembled these ceRNA interactions into 24 ceRNA networks (Supplementary Figure S4). In this study, the triplets (lncRNA-miRNA-mRNA) identified in normal tissues are not the same in cancer tissues because the ceRNA interaction were identified based on the expression of lncRNA, miRNA and mRNA for normal and tumor samples separately. The evaluation of the ceRNA networks topology reveals that the degree of these ceRNA networks all obey power law distribution, indicating that these ceRNA networks are scale free, which conform to the characteristic of biological network (Supplementary Figure S5).

**Figure 1:**
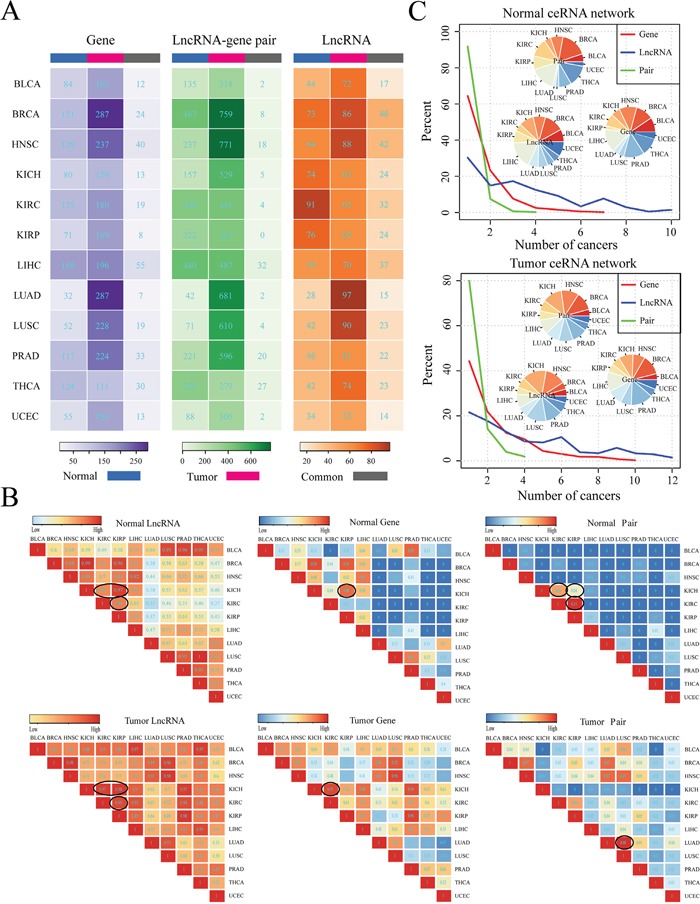
Global comparison of pan-cancer ceRNA networks **A.** Comparison of ceRNA networks within cancer types between tumor and corresponding normal ceRNA network at the lncRNA, PCG and ceRNA association levels, respectively. The number in the graph represents the count of lncRNAs/PCGs/ceRNA pairs specifically involved in the normal/tumor network or shared by the normal and tumor networks for a given cancer type. Normal (Tumor) means that lncRNAs/PCGs/lncRNA-PCG pairs that uniquely involved in the normal (tumor) ceRNA network for a given cancer type; while common represents that lncRNAs/PCGs/lncRNA-PCG pairs that involved in both of the normal and tumor ceRNA networks for a given cancer type. Here, the lncRNA-gene pairs did not consider miRNAs. **B.** The Jaccard coefficient matrix shows the similarity of ceRNA networks across 12 cancer types. Jaccard coefficients in the matrix were determined based on the shared number of lncRNAs/PCGs/ceRNA pairs between any two ceRNA networks in normal and tumor states respectively. Some pairs of cancers with same tissue of origin and relative high Jaccard coefficient within the matrix were specifically circled. **C.** Distribution of the number of cancer types that lncRNAs (blue), PCGs (red) and ceRNA pairs (green) are involved in ceRNA networks across 12 cancers. The ceRNA pairs referred to lncRNA-PCG interactions that did not involve miRNAs. The inset pie chart shows the distribution of lncRNAs, PCGs and ceRNA pairs that present in ceRNA network of only a single cancer.

CeRNA interaction of lncRNA-mRNA is not only important in the normal physiology, but also play critical role in the tumorigenesis. Moreover, different types of cancer may share some commonalities but also have cancer-specific molecular mechanisms. We thus then compared the ceRNA networks across 12 cancers and their corresponding normal ceRNA networks at the lncRNA, mRNA and lncRNA-mRNA ceRNA pair level respectively. Firstly, we compared the tumor and corresponding normal ceRNA network of each single cancer by calculating the intersection of lncRNAs, protein coding genes (PCGs), lncRNA-PCG interactions and the competing triplets in the two ceRNA networks (Figure 1A and Supplementary Table S4). These result suggest that the tumor and normal ceRNA network all exhibit great differences in 12 cancers (Figure 1A). Furthermore, we found that most of these common ceRNA pairs (lncRNA-mRNA) of were mediated by at least one same miRNAs in 12 cancers (Supplementary Table S4). We then further calculated the Jaccard coefficient for each cancer type to measure the similarity between normal and tumor ceRNA network at the lncRNA, PCG and ceRNA pair levels respectively. As a result, we found that Jaccard coefficients that calculated based on the lncRNAs involved in each ceRNA network were significantly higher than that based on PCGs (T test: P-value= 2.048e-05) and ceRNA pairs (T test: P-value= 1.232e-06) (Supplementary Figure S6). These results suggest that PCGs were more different than lncRNAs between tumor and normal ceRNA networks, which indicate that the common lncRNAs may function as different regulation roles in tumor and normal states by competing with different genes. Then, we compared the ceRNA network between two different cancers and found that cancers that with similar original tissues tend to share common lncRNAs, PCGs and lncRNA-PCG interactions in the pan-cancer ceRNA networks (Figure 1B). For example, in both the tumor and normal networks of KIRC, KIRP, KICH, the ceRNAs tend to be shared with each other. Another example is the LUSC and LUAD related lncRNA-PCG ceRNA interactions in the tumor networks. We also calculated the Jaccard coefficient of competing triplets between tumors with similar tissue origin that circled in the Normal (Tumor) pair section of Figure 1B. As shown in Supplementary Table S5, the Jaccard coefficient of competing triplets and ceRNA pairs were consistent with each other for these cancer types with similar tissue origin. This indicate that most of ceRNA pairs that shared between cancer types with similar tissue origin were mediated by at least one the same miRNAs. In addition, we found that PCGs in different cancer ceRNA networks were also more different than that of lncRNAs (Figure 1B), which indicate that some lncRNAs may regulate different PCGs in various cancers. Finally, we compared the ceRNA networks across pan-cancers and reveals that most of the lncRNA related ceRNA interactions were cancer specific and only a small fraction of ceRNAs and ceRNA interactions were shared by multiple cancers (Figure 1C). This may be due to the tissue specific expression of lncRNAs and genes and also highlight the importance of pan-cancer ceRNA interaction analysis.

### CeRNAs dominate critical cancer hallmark processes

**Figure 2:**
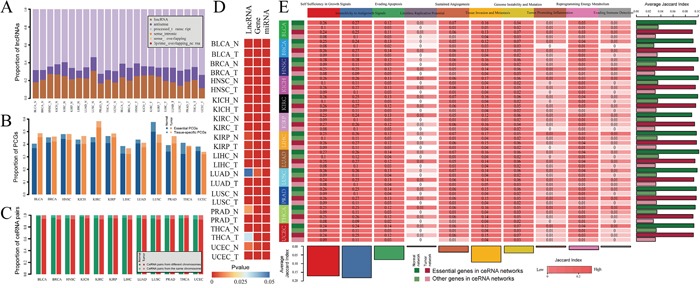
The properties of pan-cancer ceRNA molecules **A.** Proportion of lncRNAs in pan-cancer ceRNA networks that involved in each lncRNA class. **B.** Proportion of PCGs in pan-cancer ceRNA networks that are tissue specific and essential genes. **C.** Chromosome distribution of lncRNA-mRNA ceRNA pairs across 12 cancers. Dark green (light green) represent percent of ceRNA pairs in which lncRNA and PCG located in different (same) chromosome in normal ceRNA networks. Dark red (light red) represent percent of ceRNA pairs in which lncRNA and PCG located in different (same) chromosome in tumor ceRNA networks. **D.** P-value of hypergeometric test that evaluated significance of lncRNAs, PCGs and miRNAs that mediated ceRNA interactions enriched in the corresponding cancer related molecule (lncRNA/miRNA/PCG) sets. **E.** Jaccard coefficient matrix for PCGs in pan-cancer ceRNA networks and cancer hallmark processes.

In order to further understanding these ceRNA crosstalk, we examined the properties of lncRNAs and PCGs in these ceRNA networks and explored their roles in tumorigenesis. We firstly detected the class of lncRNAs in pan-cancer ceRNA networks according to lncRNA annotation from the GENCODE consortium. We found that most of these competitive lncRNAs were classified as lincRNA, antisense and processed_transcript (Figure 2A). A definite proportion (~35%-52%) of PCGs in the ceRNA networks were essential genes (Figure 2B), which suggest that these competing PCGs are fundamental importance in physiological states. We further analyzed the lncRNA-PCG (mRNA) competing pairs and found that almost all of the ceRNA pairs were located in different chromosomes (Figure 2C). This indicates that the lncRNA-mRNA ceRNA interactions tend to be distant regulation.

Next, we explored the functional roles of these ceRNAs in tumorigenesis. We firstly collected cancer related lncRNAs, genes and miRNAs from currently published data sources (see materials and method). To determine whether ceRNAs (lncRNAs and mRNAs) and miRNAs that mediated the competitive regulation are intrinsic cancer driver genes or that are closely relevant in tumors, we performed the hypergenomic test to evaluate the significance of interaction between cancer related lncRNAs, genes, miRNAs and ceRNAs and miRNAs that mediated them. Except lncRNAs in the LUAD normal ceRNA network, ceRNAs (lncRNA and mRNA) are all enriched in the cancer related lncRNAs and PCGs respectively (P-value < 0.05) (Figure 2D). In addition, miRNAs that mediated the lncRNA-mRNA competing pairs also tend to be related with cancers (Figure 2D). We then explored whether these ceRNAs for each cancer were targeting cancer hallmark processes which have been nominated that promote tumor growth and metastasis [14]. After we collected the cancer hallmark processes (see materials and method), Jaccard coefficient were used to measure intersection between cancer hallmark genes and genes in each ceRNA network. As a result, we found that genes in the ceRNA networks represented in a broad range of cancer hallmarks (Figure 2E). In particularly, hallmarks including ‘Self Sufficiency in Growth Signals’, ‘Insensitivity to Antigrowth Signal’, ‘Tissue Invasion and Metastasis’ and ‘Evading Apoptosis’ were the most four highly enriched across different cancers, suggesting that these hallmarks tend to be common in various cancers. In addition, we also found that ceRNAs that are essential genes tend to present in the cancer hallmark processes when compared with these non-essential genes across each ceRNA network (Figure 2E).

In summary, these findings provide further evidences to support that ceRNAs may not only play critical roles in normal physiological states, but is also closely related with tumorigenesis.

### LncRNAs in ceRNA networks are potential biomarkers for cancers

**Figure 3:**
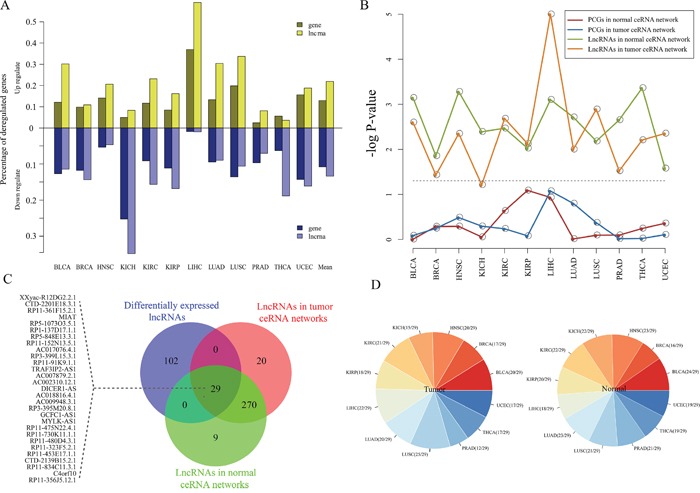
Expression analysis of pan-cancer ceRNA networks **A.** The percentages of the dysregulated lncRNAs and PCGs across 12 cancer types. Dysregulated lncRNAs and PCGs were determined by these Fold change (>2 or <1/2) by comparing normal and tumor samples for each cancer type. **B.** Statistical significance of dysregulated lncRNAs and PCGs that are enriched in ceRNA networks, which evaluated by using hypergeometric test. Gray dotted line correspond to the 5% significance level. **C.** Venn plot for dysregulated lncRNAs, and lncRNAs in the pan-cancer ceRNA networks. **D.** Distribution of these 29 shared lncRNA among three lncRNA sets in (B) in each cancer ceRNA network. The left (right) panel represents the distribution of these 29 lncRNAs in 12 tumor (normal) ceRNA networks.

Cancers are often associated the aberrant transcriptomes [15], for instance, the dysregulation of lncRNAs have been widely observed in tumors [16, 17]. In this section, we focused on exploring the dysregulation of ceRNAs in pan-cancers. We analyzed lncRNA and mRNA expression from 4515 tumors across 12 cancer types as well as 512 normal specimens from their matching cancer types in TCGA (Supplementary Table S1). For each cancer type, we extracted the differently expressed lncRNAs and mRNAs (Fold change >2 or < ½) by comparing the tumor and normal samples. To characterize cancer-associated dysregulation of ceRNA expression. For each cancers, we firstly integrated the normal and tumor ceRNA pairs and regarded them as ceRNA interactions for this cancer. Next, we examined the proportion of ceRNAs that differentially expressed in each cancer. In these 12 cancer types, the range of 3.6%~59.1% (average: 21.9%) and 1.1%~35.3% (average: 13.5%) of lncRNAs in ceRNA network significantly up- and down-regulated, respectively; while 2.5%~36.9% (average: 12.9%) and 1.0%~25.5% (average: 10.9%) of genes significantly up- and down-regulated, respectively (Figure 3A). Interestingly, the percentage of dysregulation lncRNAs were higher than PCGs in cancer ceRNA networks (Figure 3A). The hypergeometric test were carried out to further demonstrate this finding (Figure 3B). Moreover, we found that these dysregulation lncRNAs tend to be shared among different cancer types (Figure 3C–3D). These findings indicate that lncRNAs that participated in competitive regulations may be promising biomarkers in cancers.

### Network hub analysis reveals hub lncRNAs regulate variable hallmark processes across normal and cancer states

**Figure 4:**
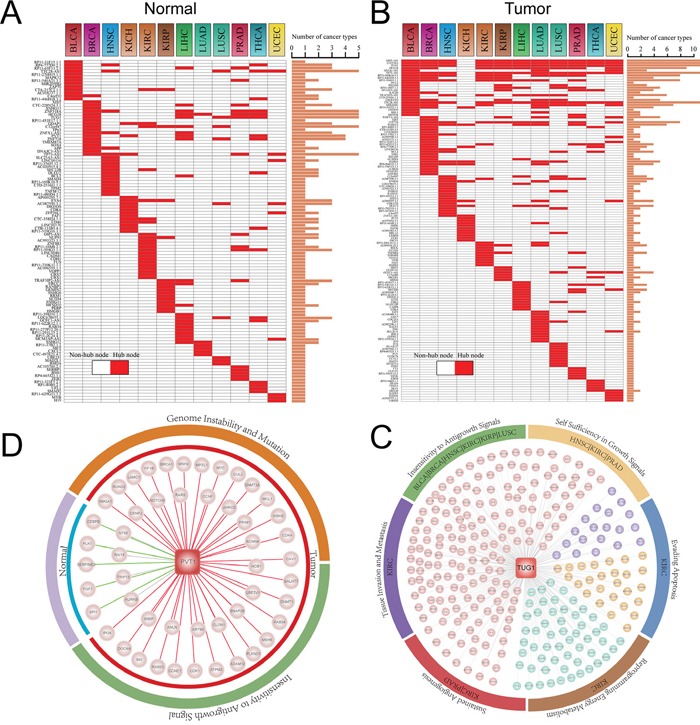
Hub analysis of pan-cancer ceRNA networks **A-B.** Distribution of hub lncRNAs and PCGs across pan-cancer ceRNA networks. Histogram represent the number of cancer types that the corresponding lncRNA (PCG) was identified as hub node in ceRNA networks. **C.** A conserved hub lncRNA TUG1 regulated variable cancer hallmark processes in different cancer types. Coloured circle represents cancer hallmarks, while the marked cancer type name indicates PCGs competitively regulated by TUG1 in their tumor networks were enriched in the corresponding cancer hallmark. **D.** A specific lncRNA PVT1 of tumor ceRNA networks regulated variable cancer hallmarks between normal and tumor state of KICH. In the tumor state it regulated Genome Instability and Mutation and Insensitivity to Antigrowth Signal, while in normal state there is no cancer hallmark was regulated by PVT1.

Hub has been known that play important roles in biological network as they have extremely high connectivity and critical for maintaining the stability of network. We thus analyzed the hubs with the top 10% highest degree of nodes in each ceRNA network [18, 19]. In total, we identified 111 hubs across 12 normal ceRNA networks and 139 hubs across 12 tumor ceRNA networks (Figure 4A–4B). We then also calculated the minimal degree value of hub nodes for each ceRNA network. As shown in Supplementary Table S6, we found that hub nodes of most of the ceRNA networks (~80%) with a node degree exceeding 5. This indicates that our definition of hub node consistent with the study of Han et al., in which they defined a hub as a node degree exceeding 5 [20]. Many of these network hubs including lncRNAs such as XIST, TUG1, PVT1, DLEU2 and H19 and genes such as SMAD4, BCL1/2 and CCND1 were highly associated with tumorigenesis. For example, Yildirim and colleagues found that lncRNA Xist is a potent suppressor of hematologic cancer in mice [21]. Another example is lncRNA PVT1 which has been demonstrated associated with multiple cancer types such as prostate cancer, lung cancer and bladder cancer [22–26]. H19 may be one of well-known lncRNAs has been demonstrate to promote cell growth and proliferation in breast and hepatocellular cancer [27]. For hub PCGs, CCND1 and SMAD4 are cancer driver genes that mediated cell cycle and TGF-β signaling which are all cancer development related biological processes [28].

Hub ceRNAs including lncRNAs and genes are competing with more ceRNAs that those non-hubs in the network. We thus infer that hub ceRNAs should have higher expression level than those non-hub ceRNAs as they should have sufficient abundance to compete with multiple ceRNAs. To confirm this assumption, we compared the expression of hub ceRNAs with that non-hub ceRNAs of each ceRNA network. The result shows that hub ceRNAs have higher expression than these non-hubs (Supplementary Figure S7-S83).

Global view of the ceRNA network hubs in pan-cancers and their normal states found that hubs are more conserved in the tumor ceRNA networks than those in normal ceRNA networks (Figure 4A–4B). Specifically, the ceRNA hubs retained their high degree in at most five ceRNA networks in normal states, while some ceRNAs can maintain their hub roles in up to 11 cancer ceRNA networks. This indicates that these conserved hubs may be maintain core skeleton of ceRNA networks in cancers. TUG1, which is such a hub lncRNAs, is identified as hub node in 11 cancer ceRNA networks (Figure 4B). Up regulation of TUG1 can promote cell growth and apoptosis in hepatocellular carcinoma [29]. Tan et al. also demonstrated that TUG1 can mediate epithelial to mesenchymal transition and radioresistance in bladder cancer cells [30]. This indicate that TUG1 may have different functions in various tumors. To explore the competing functional roles of TUG1 in different cancers, we performed the functional enrichment analysis (materials and methods) to identify TUG1 targeting hallmark processes in pan-cancers. We found that TUG1 may competitively regulate different cancer hallmarks in various cancer types. For instance, TUG1 mediated the ‘Insensitivity to Antigrowth Signals’ process in LUSC, while it mainly regulated ‘Self Sufficiency in Growth Signals’ and ‘Sustained Angiogenesis’ processes in PRAD (Figure 4C).

As shown in Figure 4A–4B, most of these hubs are specific for a certain cancer. Moreover, it has been reported that ceRNA crosstalk is important for both the physiological states and cancers [15, 31]. We thus then focused on the differential regulation of these hubs in their specific cancer type and corresponding normal state. One interesting example was a cancer associated lncRNA PVT1, which was in the top 10% of hubs in the KICH cancer ceRNA network (Figure 4B). PVT1 competitively regulated 42 and 9 mRNAs in the tumor and normal state respectively (Figure 4D). In the tumor network, PVT1 competing for cancer related genes such as BRCA1, NOTCH2 and CDK1/4. Next, we explored the variability functions of PVT1 in the normal physiological conditions and tumor. Functional enrichment analysis was carried out to identify PVT1 regulated cancer hallmark processes based on its directed connect mRNAs in normal and tumor ceRNA networks of KICH. The result suggest that PVT1 plays different functional roles in normal and tumor state. In the KICH tumor state, the competitive activity of PVT1 mainly mediated the ‘Genome Instability and Mutation’ and ‘Insensitivity to Antigrowth Signals’ hallmark processes, while no cancer hallmark process was targeted by PVT1 in the normal state.

In summary, these findings indicate that hub lncRNAs may exert differential functions across cancers and normal physiological states.

### The dynamic ceRNA interactions in normal and tumor states

**Figure 5:**
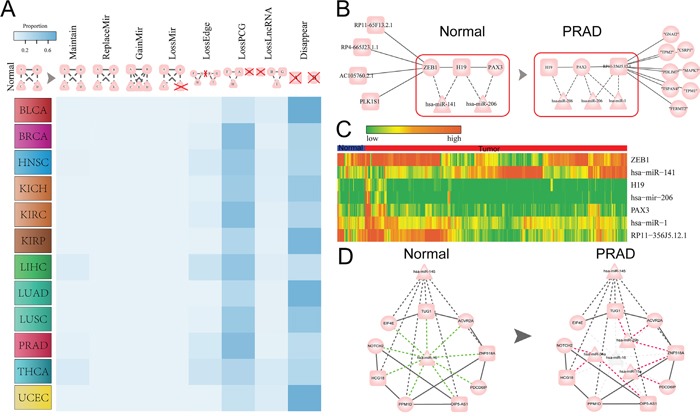
The dynamic ceRNA interactions in normal and tumor states of pan-cancer **A.** Global view of the dynamic alterations of ceRNA interactions from normal to tumor state in 12 cancers. Frequency of various alteration patterns from normal to tumor state. **B.** Dynamic regulation of H19 which is a cancer related lncRNA between normal and prostate cancer (PRAD). **C.** The expression heat map of lncRNAs, PCGs and miRNAs that mediated the ceRNA interaction in the red rectangle region of (B) across tumor (red) and normal samples (blue) of PRAD. In the heat map, highly expression are shown in red, low expression are shown in green. **D.** The conserved ceRNA subnetwork (ceRNA interactions presented in both normal and tumor networks of PRAD) between normal and tumor ceRNA network of PRAD, which shown that hsa-miR-16 was replaced by three miRNAs including hsa-miR-34a, hsa-miR-29b and hsa-miR-15a in the tumor state.

As above we have found that lncRNA may regulate different functions in normal conditions and tumors. Next, to further explore the dynamic alteration of ceRNA interactions between normal and tumor states, we consider eight possible instances of ceRNA interaction (lncRNA-mRNA) alteration between these two states (Figure 5A). Taken the ceRNA interaction between lncRNA A and PCG B in normal network as example, the eight alteration patterns including: ‘Maintain’: interaction between A and B were also involved in tumor network of the given cancer type and also mediate by the same miRNA(s) as in the normal network; ‘ReplaceMir’: interaction between A and B were also involved in tumor network of the given cancer type and mediate by the same number of miRNA, but replaced by different miRNA(s); ‘GainMir’: the number of miRNAs mediated A and B in tumor is increased; ‘LossMir’: the number of miRNAs mediated A and B in tumor is decreased; ‘LossEdge’: lncRNA A and PCG B are not interacted with each other in tumor, but they interact with other PCG(s) and lncRNA(s) respectively; ‘LossPCG’: PCG B is not present in tumor network, while LncRNA A interact with other PCG(s) in tumor; ‘LossLncRNA’: lncRNA A is not present in tumor network, while PCG B interact with other lncRNA(s) in tumor; ‘Disappear’: both lncRNA A and PCG B are not present in tumor network. We then calculated the alteration frequency of each instance in pan-cancers (Figure 5A). We found that most of the ceRNA pairs in the normal state will not be present in the tumor state (Figure 5A). In generally, lncRNAs will compete with different PCGs in the tumor ceRNA network or both of the lncRNA and gene that interact with each other in normal network will disappear in the tumor network across 12 cancer types. This indicate that a wide range of dynamic change of ceRNA interaction exist between normal physiological and tumor states, which is consistent with the viewpoint of Karreth et al. that perturbations of functional interactions in ceRNA networks will contribute to disease pathogenesis [15].

To exemplify how the dynamic changes of these competing interactions can provide insight into the pathogenesis of tumors, the ceRNA networks associated with PRAD were examined. H19 may be one of the earliest identified cancer lncRNAs, the up-regulation of H19 could repress cell migration of prostate cancer [32]. In the normal state, H19 compete with ZEB1 and PAX3, while the interaction between H19 and ZEB1 is ‘switch off’ in the tumor. At the same time, another partner of H19, PAX3 interact with a new ceRNA RP11-356J5.12.1 in tumor state (Figure 5B). We then explored miRNAs that mediated lncRNA-mRNA competitively regulation. MiR-206 mediate the H19-PAX3 competing interaction in both normal and tumor states, while miR-1 joint with miR-206 mediate RP11-356J5.12.1-PAX3 ceRNA pair in tumor (Figure 5B). Down-regulation of miR-1 could promote prostate cancer bone metastasis [33] and Hudson et al. nominate miR-1 is a candidate tumor suppressor and prognostic marker in human prostate cancer [34]. The expression levels of miRNAs have been demonstrated that critical for the ceRNA activity [35], we thus then examined the expression of miRNAs mediate the dynamic regulation of ceRNAs. Interestingly, we found that miR−141 (mediate ceRNA activity of H19-ZEB1 in normal) with relative low expression in normal state. And miR−206 (mediate ceRNA activity in both normal and tumor) always keep a relative low expression level in both normal and tumor state (Figure 5C and Supplementary Figure S9) compared with miR−141 and miR−1. This may consistent with the findings that the concentrations of miRNA and ceRNA related with ceRNA activity in the study of Ala et al. [35]. Furthermore, except the concentration of ceRNAs and miRNAs that mediated them may impact the ceRNA activity, another important mechanism of a switch off of ceRNA is the exon skipping mechanism or the length of 3′UTR as discussed in the study of Paci et al. [12]. We found that H19 has 13 alternative transcripts and some of them do not harbor the seed matches of miR−141. Thus, the observed ‘switch off’ in tumor of the H19-ZEB1 ceRNA activity may be due to the skipping of the exons where the MREs reside. By exploring the expression of the competing triplets H19-miR-206-PAX3, we also found that the correlations between miR-206 with the two ceRNAs (H19 and PAX3) were all positive in normal and tumor states. This may due to that there is a “MIXED-sponge” motif that reported in the study of Paci et al. [12] about this competing triplet.

Next, we focused on these maintained ceRNA interactions between normal and tumor ceRNA networks of PRAD. We found that a ceRNA module which mediated by hsa-miR-145 and hsa-miR-16 in normal state, while the hsa-miR-16 was replaced by three other miRNAs including hsa-miR-29b, hsa-miR-34a and hsa-miR-15a to maintain these original ceRNA interactions in normal state (Figure 5D). It is notable that all of these three replaced miRNAs in tumor state are relevance in the tumorigenesis of prostate cancer. The study of Ru et al. reported that miRNA-29b suppresses prostate cancer metastasis by regulating epithelial-mesenchymal transition signaling [36]. MiR-15a could mediate the cross-talk between tumor and microenvironment in prostate cancer [37]. MiR-34a can repress prostate cancer stem cells and metastasis through targeting CD44 [38]. Overall, these observations highlight the importance of analyzing dynamic regulation of ceRNA interactions to explore the mechanism of tumorigenesis.

### Network analysis reveals miRNA-mediate ceRNA regulated pattern in pan-cancers

**Figure 6:**
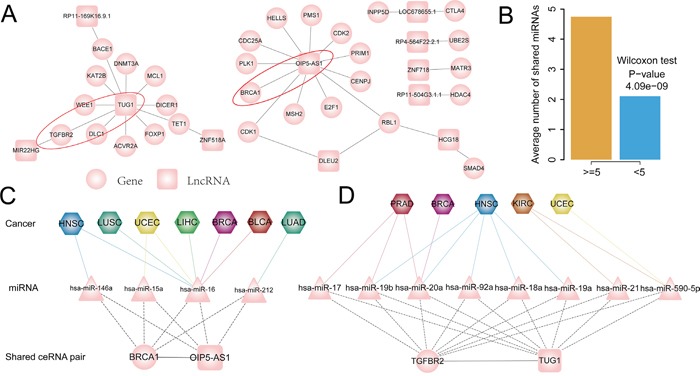
**A.** Conserved ceRNA subnetwork of pan-cancer, in which ceRNA pairs presented in tumor ceRNA network of more than five cancer types. **B.** Conserved ceRNA pairs shared more miRNAs than others. **C.** OIP5-AS1-BRCA1 ceRNA pair presented in seven cancers and mainly mediated by hsa-miR-16. **D.** TUG1- TGFBR2 ceRNA pair present in five cancers and it is mediated by different miRNAs in different cancer types. Circular nodes represent PCGs in ceRNA network, colored rectangle represent lncRNAs, triangle correspond to miRNAs and hexagon represent cancer types.

Different cancers have common biological characters such as proliferation and metastasis, we thus aim to understanding these commonalities among various cancers underlying the ceRNA interactions context. We firstly examined whether there is a core ceRNA interactions that shared by different cancers, we extracted ceRNA pairs that presented in at least five cancer ceRNA networks. In total, 33 ceRNA interaction pairs were obtained and then assembled these interactions into a subnetwork (Figure 6A). Several cancer related PCGs were involved such as CDK1/2, BRCA1 and TGFBR2, suggesting that lncRNAs in the core subnetwork may mediate the tumorigenesis by competitively regulating these cancer driver genes in pan-cancers. The above observations suggest that these ceRNAs might mediate the development of various tumors through similar mechanism.

Previous studies have indicated that the density of miRNA binding site on ceRNAs may affect competing activity. Based on this concept, we assume that ceRNA pairs that presented in multiple caner types will share more microRNAs as they would be expected to have a higher probability of forming ceRNA pairs. Next, we explored miRNAs that mediated these common ceRNA pairs across cancers. We found that the number of shared miRNAs between conserved ceRNA pairs is significantly higher than that of cancer-specific ceRNA pairs (Wilcoxon test: P-value= 4.09e−09; Figure 6B). To examine how miRNAs mediate these conserved ceRNA pairs in pan-cancers and to gain insight into the commonalities of various cancers, we focused on two ceRNA pairs: OIP5-AS1-BRCA1 and TUG1-TGFBR2, both of these two mRNAs are cancer driver genes and lncRNAs with highly degree in the subnetwork. OIP5-AS1-BRCA1 ceRNA pair present in seven cancer ceRNA networks and mediate by four miRNAs including hsa-miR-146a, hsa-miR-15a, hsa-miR-16 and hsa-miR-212 (Figure 6C). Further exploring the ceRNA pairs across cancers found that hsa-miR-16 mediate the ceRNA pair in up to six of cancer types, while hsa-miR-146a, hsa-miR-15a and hsa-miR-212 coordinately mediate this ceRNA pair with hsa-miR-16 in HNSC, UCEC and LUAD respectively (Figure 6C). This indicate that the ceRNA activity of some lncRNA-mRNA pair may be mediated by the same miRNA across cancers. In contrast to OIP5-AS1-BRCA1 ceRNA pair, which is mediated by the same miRNA in multiple cancers, TUG1-TGFBR2 ceRNA interaction is mediated by different miRNAs in five cancers (Figure 6D). For example, hsa-miR-92a, hsa-miR-18a and hsa-miR-19a mediate the TUG1-TGFBR2 interaction only in the HNSC, while hsa-miR-21 specifically mediate this ceRNA pair in the KIRC. Taken together, these results indicated that some miRNAs might selectively mediate ceRNA interactions in a specific cancer, while some might mediate the same ceRNA interaction in pan-cancers.

### Cancer-specific ceRNA interactions mediate key biological functions

**Figure 7:**
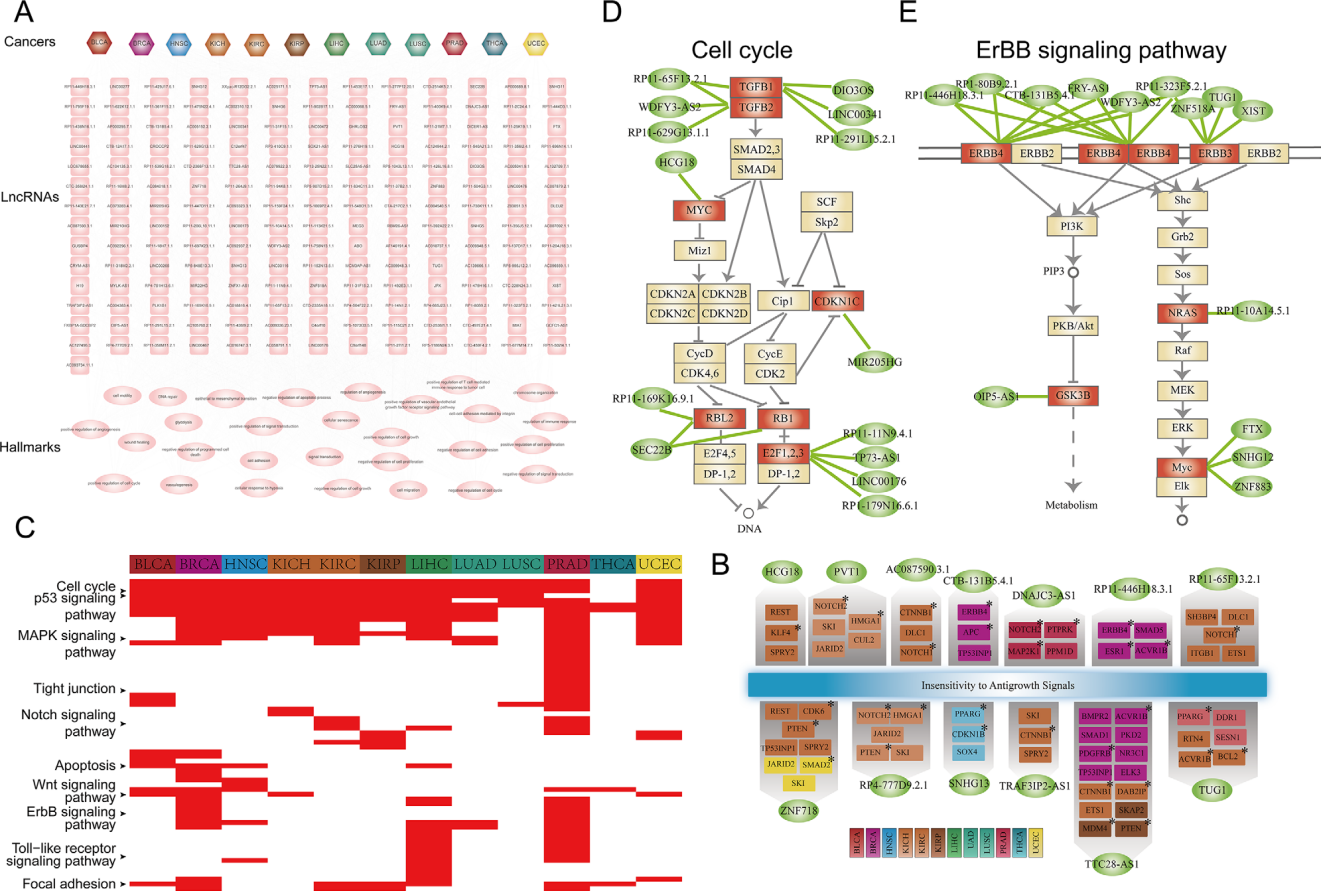
Functional analysis for lncRNAs that participate in cancer specific ceRNA interactions **A.** Thecancer-lncRNA-hallmark hierarchical network. An edge between a cancer node and lncRNA node represent the lncRNA participates specific ceRNA pair(s) of the corresponding cancer type. An edge between lncRNA and cancer hallmark represent PCGs that specifically competing with the given lncRNA were enriched in the cancer hallmark process (P<0.05). Hexagon nodes represent cancer, rectangle nodes represent lncRNA and ellipses nodes are cancer hallmarks. **B.** Thirteen lncRNAs in (A) regulated ‘Insensitivity to Antigrowth Signals’ hallmark process through distinct genes in different cancers. Protein coding genes (PCGs) were colored according cancer type in which it specifically interacted with the corresponding genes. Cancer PCGs were marked by star. Ellipses nodes with green color represent lncRNAs, rectangle nodes represent PCGs. **C.** Pathway enrichment analysis for PCGs involved in cancer specific ceRNA pairs. In the heat map, the corresponding cell was colored red if PCGs involved in this cancer type specific ceRNA pairs were significantly enriched in the pathway. **D.** LncRNAs cooperatively regulated the cell cycle pathway in PRAD. Ellipses nodes with green color represent lncRNA, rectangle nodes with red color represent genes were competitively regulated by lncRNAs, while yellow node represent pathway genes. **E.** Similar with (D), but ErBB signaling pathway for BRCA.

We have observed in the above analysis that most of ceRNA interactions are cancer-specific, we then explored the functional roles of lncRNAs that participate cancer-specific ceRNA interactions in tumorigenesis. Functional enrichment analysis were carried out for coding genes of each lncRNAs based the cancer hallmark processes. Firstly, we found that processes including ‘cell proliferation’, ‘cell growth’, ‘cell apoptosis’ and ‘cellular response to hypoxia’ were associated with most of these lncRNAs in different cancers. This indicate that these functions which are important for the initiation and progression of cancer will be activated by the ceRNA competitive regulation in pan-cancers. Some well-known cancer related lncRNAs such as H19, PVT1 and XIST all regulated the ‘negative regulation of cell proliferation’ process across different cancers (Figure 7A). This suggest that these cancer lncRNAs regulated different coding genes to competitively mediate the same cancer hallmark process. We then examined the ‘Insensitivity to Antigrowth Signals’ hallmark class which is the most highly enriched by PCGs in pan-cancer ceRNA networks. We found that lncRNAs competitively regulated many cancer genes in different cancers (Figure 7B). For example, PVT1 regulated NOTCH2 and HMGA1 in KICH. NOTCH signaling has been known that play important role in cell-fate determination, differentiation and proliferation of tumorigenesis [39]. The study of Takaha et al. suggest that HMGA1 is a potential target for novel therapeutic modalities for metastatic renal cell carcinoma [40]. TUG1 regulated the cancer genes ACVR1B and BCL2 in KIRC, while it competitively regulated PPARG in BLCA (Figure 7B). Then, the KEGG pathway enrichment analysis were performed for all protein-coding genes that participate in these cancer specific ceRNA interactions (see methods). Many cancer related biological pathways such as ‘cell cycle’, ‘P53 signaling pathway’, ‘MAPK signaling pathway’ and ‘ERBB signaling pathway’ were enriched for these genes (Figure 7C). Next, to exemplify how lncRNAs that participate cancer specific ceRNA interactions that synergistically regulated genes in these key biological pathways, the ‘cell cycle’ and ‘ERBB signaling pathway’ were examined. In prostate cancer, six lncRNA (LINC00341, RP11-291L15.2.1, DIO3OS, RP11-65F13.2.1, WDFY3-AS2 and RP11-629G13.1.1) cooperatively regulated TGFB which is critical for the development of tumor (Figure 7D). Another example is the ‘ERBB signaling pathway’ for breast cancer, which is a hallmark signaling for the initiation and progression of breast cancer. ERBB3 is the upstream gene of this pathway, which is collectively regulated by four lncRNAs including XIST, TUG1, ZNF518A and RP11-323F5.2.1 (Figure 7E). This indicate that lncRNAs in the cancer ceRNA network synergistically regulated key genes in cancer related biological pathways.

### CeRNA crosstalk modules may be potential biomarkers for cancer prognosis

**Figure 8:**
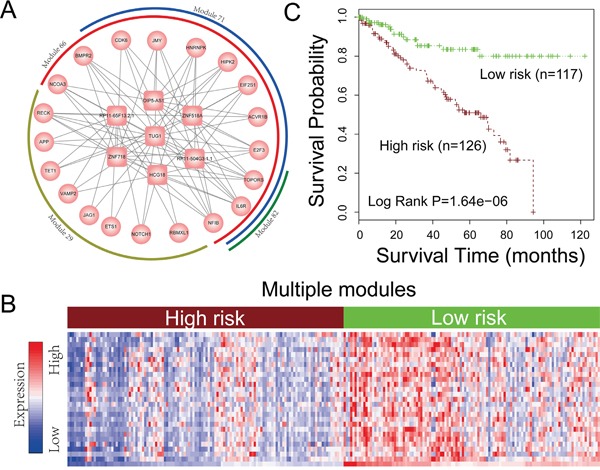
Network module analysis for ceRNA network **A.** Four ceRNA modules of KIRC crosstalk with each other. The rectangles represent lncRNAs and circles represent protein coding genes. **B.** Hierarchical clustering of 255 patients based on expression of lncRNAs and PCGs in (A) The rows of the clustering are all the ceRNAs involved in Figure 8A and columns of the clustering are KIRC samples. **C.** Kaplan-Meier survival analysis of two groups of patients in (B) with different clinical outcomes. The Kaplan-Meier curves are performed based on all the ceRNAs (lncRNAs and PCGs) in the four crosstalk modules of Figure 8A.

The lncRNA related ceRNA networks provide a global landscape of the competitively regulation in pan-cancers. However, network modules, which are a subset of ceRNAs that closely connected with each other in the network, can provide more detailed information about ceRNA regulation in pan-cancers. We thus then extensively identified the network modules across 12 cancer ceRNA networks (see materials and methods). In total, 4946 ceRNA modules were identified. Based on the notion that the prediction power of survival of a module biomarker is better than that of an individual gene [41], we therefore evaluate the potential ability of these ceRNA modules for prediction of the prognosis of cancer. As a result, 1196 ceRNA modules can be used to classify cancer samples into two groups with significantly different overall survival rates (log-rank test, P< 0.05). In particularly, we found four representative prognostic modules (module 29, module 66, module 71 and module 82) that crosstalk with each other in KIRC could distinguish patients with different clinical outcomes (Supplementary Figure S10). As these four modules were crosstalk with each other (Figure 8A), we then explored the prediction power of the ceRNA crosstalk module which including module 29, module 66, module 71 and module 82. The result indicates that the ceRNA crosstalk modules can not only stratify the patients into groups with significant different survival rates (Figure 8), but also could improve the prediction power when compared with using single module (Figure 8; Supplementary Figure S10). By using the risk score model (Materials and Methods) to reclassify patients into two groups, this crosstalk module was also significantly associated with survival (P = 5.23E−5 in Supplementary Figure S11). A further exploration of this ceRNA crosstalk module found that many protein-coding genes in it were associated with human cancers such as CDK6 and NOTCH1. For example, CDK6 plays critical roles in regulating the progression of cell cycle and have been recently demonstrated to have a transcriptional role in tumor angiogenesis [42]. The activation of notch1 could promote renal cell carcinoma growth via PI3K/Akt signaling [43]. In summary, all of the above indicate that the ceRNA crosstalk modules may have the potential for prediction of cancer prognosis.

## DISCUSSION

The last few years have nominated ceRNA hypothesis as a novel layer of gene regulation. Several studies have suggest that lncRNA could serve as miRNA sponges by decoying miRNAs from other target transcripts and thus play critical roles in the development of cancer. For example, lncRNA HOTAIR could function as a competing endogenous RNA to regulate HER2 expression by sponging miR-331-3p in gastric cancer [44]. The study of Liang *et al.* have demonstrated that lncRNA H19 could induce epithelial to mesenchymal transition by functioning as miRNA sponges in colorectal cancer [4]. However, our knowledge about the molecular mechanism of lncRNA that act as ceRNAs in tumor is still limited. In this study, we systematically constructed and dissected the lncRNA related ceRNA networks in pan-cancer by integrating multiple level molecular profiles of large-scale samples and miRNA regulation, which will lead significant insight into understanding the novel aspect of gene regulation involved non-coding RNAs in human cancers.

The competition events of ceRNAs also generally occurred in normal physiological states. For example, lncRNA lincMD1 which is activated on myoblast differentiation and controls muscle differentiation in human and mouse myoblasts by functioning as ceRNA [5]. In addition to the alteration of the genome such as amplifications, deletions, mutations and epigenetic modifications could result in the initiation and development of disease, aberrant changes in ceRNA regulation may also contribute to disease pathogenesis. Comparison analysis of the ceRNA activity between normal and tumor states could thus help for further understanding the underlying mechanism of ceRNA activity in tumorigenesis. As a result, we found the ceRNA regulation varied greatly from normal to tumor states, which indicate that alterations of the ceRNA regulation may be a basis molecular level change in human tumor. The ceRNA molecules including lncRNAs and mRNAs were more conserved in the pan-cancer tumor networks, while the lncRNAs tend to be shared among the tumor network. This suggest that there may be a basic lncRNA related ceRNA network structure maintain the general cancer pathophysiologic processes. Higher similarity of the ceRNA interactions were observed in cancers with similar tissue of origin, which provided further evidence that the lncRNA related ceRNA regulation may be a fundamental layer of gene regulation in tumorigenesis.

In our current study, we have particularly focused on the cancer related characteristics of the ceRNA molecules in the pan-cancer networks. We found that lncRNAs and genes were enriched in the cancer gene and lncRNA sets, and a significant proportion of genes were essential genes. This indicate that molecules in the pan-cancer ceRNA networks are functional importance in both physiological conditions and are also crucially relevant in various cancers. Cancer hallmark functional analysis reveals that these ceRNA molecules were related with hallmark processes such as ‘Self Sufficiency in Growth Signals’, ‘Insensitivity to Antigrowth Signals’, ‘Tissue Invasion and Metastasis’ and ‘Evading Apoptosis’. These function are all the cell growth and apoptosis associated processes, which suggest that ceRNAs usually participate in fundamental functions involved in cancer biology.

For identifying the pan-cancer ceRNA pairs, we employed an integrated pipeline that simultaneously consider the sequence information and the expression of the ceRNAs and their shared miRNAs. In order to guarantee high confidence of the putative ceRNA pairs, only experimentally verified miRNA-target data source were used in the present study. Furthermore, other miRNA-target data source that obtained by reliable prediction algorithm such as targetScan [45] and data source that involved comprehensive information such as MirWalk [46] can also be integrated. Due to the lack of miRNA-lncRNA interaction data sources, we used the miRNA-target gene prediction algorithm combined with AGO-CLIP data to identify reliable miRNA binding sites and predicting miRNA targets. Additionally, expression factor was also considered to filter the ceRNA pairs. Due to the TCGA publication embargo, we took only the level 3 of RNA-seq V2 data for tumor types without publication restrictions. In the RNA-seq V2 level 3 data, we only can directly obtained expression for a small number of lncRNAs. Thus, to obtain a relative more lncRNA expression data, we recalculated the RPKM values for the protein coding genes and lncRNAs based on raw read counts for each exon were derived from exon quantification files provided by the TCGA level 3 data set. This strategy has been successfully used in our previous studies [10, 47] to extract lncRNA/coding genes expression from TCGA level 3 data. Specifically, in the study of Wang et al [10], they have demonstrated the accuracy of this recalculated RPKM values through comparing data obtain by this strategy with publication data. Exploring the expression of ceRNAs, we found that ceRNAs were usually with highly expression level (Supplementary Figure S12-S13) which is consist with the study of Denzler et al [48]. The expression of hub nodes were higher than others in the pan-cancer ceRNA networks (Supplementary Figure S7-S8). Indeed, hub ceRNAs are expected to have sufficient abundance to compete with their large amount of neighbors. Various factors such as the expression level of miRNAs and ceRNAs, the number of shared miRNAs have been suggested to contribute to ceRNA effectiveness. Future work may consider more factors to precisely identify ceRNA pairs and provide better understanding of ceRNA activity in human cancers.

In Paci et al.'s work [12], they identified the ceRNA pairs mainly depended on the expression data, although the sequence matched information were also considered. Thus, Paci et al.'s method tend to unbiased and could identified more novel ceRNA interactions. While, in our study, we paid more attention to the data reliability and simultaneously considered the specific expression data. Our method also depended on the different expression data of different cancer types under tumor and normal states respectively. Besides, many recently studies also used similar strategy to identify ceRNA pairs [10, 49, 50].

In the comparison of ceRNA network between normal and tumor conditions of the same cancer type, it would be better to considering only common patients as the obersvation of difference using all tumor samples may due to more statistics available for cancer samples than normal ones. We thus then rebuild these ceRNA networks across 12 cancer types based on common patients (patients with cancer and matched-normal tissues). Furthermore, retained the expression profiles for lncRNAs, miRNAs and mRNAs that RPKM >0 across more than 90% samples in each cancer type. The details of the samples and network information were shown in Supplementary Table S1 and Supplementary Table S7. The results shown that findings about comparsion of ceRNA nerworks between normal and tumor states based on sample matched data were consistent with our previous results. Specifically, in the sample-matched analysis of ceRNA networks, we also found that the tumor and normal ceRNA network all exhibit great differences in 12 cancers, and comparing with PCGs and ceRNA pairs, lncRNAs in ceRNA networks were also tend to be shared by normal and tumor ceRNA networks (Supplementary Table S7). In addition, the results of dynamic analysis based on sample matched data (Supplementary Figure S14) were also consistent with that of Figure 5A. These results suggest that considering common patients or not have little effect on the main observations about changes of ceRNA interactions between normal and cancer conditions in the manuscript. This may due to that we identified the significant ceRNA pairs based on the top rank 5% cutt-offs. This rank based strategy may reduce the statistics effects on the difference between normal and tumor ceRNA networks to some extent.

Based on reviewing previous publication reports, we found that some of known ceRNA interactions which are present in these experimental study results were involved in the pan-cancer ceRNA interactions. For example, TUG1-hsa-miR-26a-PTEN, which is a competing triplet that involved in the BRCA tumor state, has been demonstrated that TUG1 could serve as a miR-26a sponge and then contribute to the up-regulation of PTEN in human glioma cells [51]. The study of Du et al. demonstrated the tumour-suppressive function of TUG1 and its regulation of PTEN expression in prostate cancer by analyzing a long noncoding RNA-mediated sponge regulatory network in prostate cancer [49]. TUG1 has been validated by many studies that it play important roles in tumorigenesis [29, 30] and PTEN is a well-known tumor driver gene. This indicate that the competing triplets TUG1-hsa-miR-26a-PTEN may also play important roles in the initiation and progression of BRCA. Another example is TUG1-hsa-miR-34a- VEGFA, which is a competing triplet that involved in the KIRC tumor state. The study of Dong et al. have demonstrated that TUG1, miR-34a-5p, and VEGFA constitutes to a regulatory network, and participates in regulating hepatoblastoma cell function, tumor progression, and tumor angiogenesis [52]. In addition, it has been reported that H19 can function as a molecular sponge of let-7 and H19/Let-7/IGF1R regulatory pathway may related with impaired endometrial preparation and receptivity for pregnancy in women with endometriosis [53]. Interestingly, we found that H19/hsa-let-7b/IGF2BP1 competing triplet involved in the tumor state ceRNA network of UCEC. This indicates that IGF2BP1 may be a novel downstream regulation target of H19/Let-7 in UCEC.

Currently, there are many studies focused on the ceRNA crosstalk and thus provided a deeper understanding of the gene regulation. StarBase [9] and DIANA-LncBase [8] integrates the AGO CLIP-Seq experimental data to identify miRNA-lncRNA interactions, which provide useful data sources for investigating ceRNA regulation in human diseases. Sumazin et al. constructed and dissected the glioblastoma related RNA-RNA crosstalk network [11]. Paci *et al.* had identified a sponge interaction network between long non-coding RNAs and messenger RNAs in human breast cancer [12]. A long noncoding RNA-mediated sponge regulatory network were identified in prostate cancer by the study of Du et al. [49]. All of the above studies focused on only one cancer type. Pan-cancer studies could provide a system-level insight into the ceRNA regulation mechanism in human cancers. Two such representations are our recently studies which devoted to investigate the ceRNA crosstalk in pan-cancers. Xu et al. analyzed the mRNA-related ceRNA crosstalk in 20 major cancers [50]; while Wang et al. focused on constructed the lncRNA related ceRNA networks which provided a valuable data source for lncRNA studies [10]. However, in our current studies, we mainly focused on comprehensively characterizing the lncRNA related ceRNA network across 12 cancer types and the corresponding normal states. Especially, we revealed many previously un-characterized important aspects of ceRNA crosstalk in cancers by comparing the ceRNA regulation within and cross different cancer types and ceRNA network dynamic analysis.

In summary, our study present the lncRNA related ceRNA crosstalk landscape across pan-cancers and normal states, which offers new avenues for examining the perturbation that contribute to cancer pathogenesis. By systematically analyzing the ceRNA networks, we reveal some important properties of ceRNA regulation. These findings provide new insight into understanding the gene regulation mechanism in human cancers and help to facilitate a variety of future studies such as discovery of potential prognostic biomarkers and therapeutic targets.

## MATERIALS AND METHODS

### The candidate lncRNA-miRNA-mRNA competing interactions

The lncRNA-miRNA-mRNA interactions data were obtained from our previous study in which we developed a pipeline to identify lncRNA associated competing triples [10]. In brief, we firstly predicted the miRNA-lncRNA interactions based on four miRNA target prediction methods including miRanda, RNAhybrid, TargetScan and PITA. The miRNA-lncRNA interactions predicted by different methods were integrated. Then, the Argonaute-CLIP data were used to filter the miRNA-lncRNA interactions to identify experimentally supported miRNA-binding sites on lncRNA sequences. The miRNA-mRNA interactions were obtained from two high-quality databases including TarBase and mirTarBase. LncRNA-mRNA pairs that shared one miRNAs were identified as one candidate lncRNA-miRNA-mRNA competing interaction. Finally, we got 526173 non-redundant lncRNA-miRNA-mRNA interactions for further analysis.

### The sample matched normal and tumor expression data in 12 cancers

We downloaded the miRNA (IlluminaHiSeqmiRNASeq) and RNA (IlluminaHiSeqRNASeqV2) level 3 expression data of 12 cancers from TCGA database (version April, 2015, Supplementary Table S1) through the Data portal [54]. The clinical information were also obtained for further analysis.

We extracted the lncRNAs and protein coding genes (PCGs) expression data of 12 cancers from the raw read counts of each exon. The exon counts data were obtained from exon quantification files provide by the TCGA level 3 RNASeqV2 dataset. Then, we recalculated the RPKM expression values of lncRNAs and mRNAs in each sample at according to our previous study [10]. The detailed calculation formula was as follows: RPKM = (raw read counts ×10^9) / (total reads ×length of lncRNA/coding genes); in which the raw read counts=sum of raw read counts in all exons mapped entirely within the lncRNA/coding gene loci; total reads=sum of raw read counts calculated for all exons of a single sample. Finally, the matched lncRNA and mRNA expression data of 4515 tumors and 512 normal samples were obtained across 12 cancers.

### Collection of cancer related lncRNAs, protein coding genes and miRNAs

In order to explore the functional roles of these ceRNAs in tumorigenesis, we examined that whether ceRNAs involved in the pan-cancer ceRNA networks and miRNAs that mediated these pan-cancer ceRNA activity are intrinsic cancer driver genes or that are closely relevant with tumors. Thus, we collected the cancer related lncRNAs, protein coding genes and miRNAs sets. The cancer associated lncRNAs were derived from LncRNADisease [55]. We collected the cancer related genes from COSMIC [56] and the study of Tamborero et al. [57], which are aim to identify the cancer driver genes. For miRNAs that related with cancer were extracted from HMDD [58] and miR2Disease [59], both of these are all manually curated databases for microRNA deregulation in human disease. In total, 53 lncRNAs, 1046 genes and 249 miRNAs that associated with cancer were obtained. Then, we used the hypergenomic test to evaluate whether these ceRNAs in pan-cancer ceRNA network and miRNAs that mediated their interaction were significantly enriched in our collected cancer related lncRNAs, protein coding genes and miRNAs sets.

### Essential genes and tissue-specific genes

In order to dissect the properties of ceRNAs, we then explored these protein coding genes in pan-cancer ceRNA networks tend to be essential genes or tissue-specific genes. Essential genes and tissue-specific genes we collected in this study are all protein coding. The essential genes were obtained from our previous study [60], in which essential genes were collected by using the phenotype information of the corresponding mouse orthology. Briefly, if a mouse suffered from the lethality when a particular gene was knocked out, a human ortholog of this gene was defined as an essential gene. In total, 2486 mouse lethal human orthologs were identified as human essential genes. Tissue-specific protein coding genes were obtained from the study of Chang et al. [61], in which these genes were systematically identified from the gene expression profiles across 43 normal human tissues. In their study, they adapted the tissue-selective score developed in a previous study [62] to identify tissue-specific genes. In total, 2293 tissue-specific protein coding genes were obtained. Then, we calculated the proportion of essential genes and tissue-specific genes involved in each ceRNA networks.

### Cancer hallmarks for functional analysis

The cancer hallmark Gene Ontology (GO) terms were derived from a previous study [63]. Then, genes that annotated in these hallmark GO terms were obtained from MsigDB database which collected the GO term functional set for GSEA analysis [64].

### Construction of the lncRNA related ceRNA network for each cancer

For each cancer type, we constructed the lncRNA related ceRNA crosstalk network by integrating the matched lncRNA, miRNA, mRNA expression profiles and the candidate 526173 lncRNA-miRNA-mRNA competing interactions which is obtained from our previous work in [10] (see ‘The candidate lncRNA-miRNA-mRNA competing interactions’ section). First, we filtered the expression profile for lncRNAs, miRNAs and mRNAs that with RPKM >0 across more than 50% samples in each cancer type were retained for further analysis. Then, for each candidate lncRNA-miRNA-mRNA interaction, we identified the lncRNA-mRNA as ceRNA pair as they satisfied the following two criteria simultaneously (Supplementary Figure S1): (i) The role of miRNA in mediating correlation of lncRNA and mRNA should be significant; (ii) the expression of lncRNA and mRNA should significantly positively correlate with each other. We used the Sensitivity Correlation of Paci et al. [12] to evalue the strength of miRNA in mediating correlation of lncRNA and mRNA and Pearson correlation coefficient to measuring the correlation between lncRNA-mRNA pairs. In this study, in order to ensure that more possible ceRNA interactions were considered and at the same time make the number of false positives within an acceptable range, we chose top 5% as signifcant threshold.

To infer the significance role of miRNA that mediate the correlation of lncRNA and mRNA for each candidate lncRNA-miRNA-mRNA interaction, we performed the partial correlation analysis which used in the study of Paci et al. [12]. For example, we take L-Z-M represents a candidate lncRNA-miRNA-mRNA interaction. The calculated formula were as follow:

$$R_{\text{ML}|Z}=\frac{R_{\text{ML}}-R_{\text{MZ}}R_{\text{ZL}}}{\sqrt{1-R_{\text{MZ}}^{2}}\sqrt{1-R_{\text{ZL}}^{2}}}$$ (1)

Where, *R_ML_*, *R_MZ_*, *R_ZL_* represent the Pearson correlation coefficient between mRNA and lncRNA, mRNA and miRNA, miRNA and lncRNA in the given lncRNA-miRNA-mRNA interaction respectively. Then, the Sensitivity Correlation of Paci et al. [12] of miRNA (Z), which is referred as S, for the corresponding candidate ceRNAs M and L is calculated as:

$$\text{S}=R_{\text{ML}}-R_{\text{ML}|\text{XZ}}$$ (2)

For identifying the significant correlation that satisfy (i), we firstly constructed a random background distribution of the Sensitivity Correlation of Paci et al. [12], which is defined as score S. The random background distribution of S was generated by calculating the score S of randomly selected combination of lncRNA-miRNA-mRNA competing interactions. Then, we defined the threshold for “significant” correlation as the minimal value that ranked in the top 5% of the distribution of the S values. If the score S for the observed candidate lncRNA-miRNA-mRNA competing interactions higher than the defined threshold were regarded as significant correlation.

CeRNAs such as lncRNAs can sequester the free miRNA molecules from their repressing target mRNAs. This indicate that the expression pattern of ceRNA pairs may exhibit positively correlation. We thus further required that the lncRNA-mRNA ceRNA pairs should be positively correlated with each other (i.e. satisfy (ii)). In this study, the “significantly positively correlate” refers to that the pearson correlation coefficient between the corresponding lncRNA and protein coding gene was positive (>0) and also ranked in the top 5% of the background correlation coefficient list (i.e. for all pairs of lncRNA and protein-coding gene that with positive correlation coefficient).

In summary, for a given lncRNA-miRNA-mRNA interaction L-Z-M, if the score S of miRNA (Z) and the expression correlation of lncRNA (L) and mRNA (M) satisfy the above conditions respectively, the L-M will be identified as a ceRNA pair. We then assembled all the identified lncRNA-mRNA ceRNA pairs and generated the lncRNA related ceRNA network with lncRNA and mRNA as nodes and connected if the expression of them were significantly mediated by miRNAs and they positively co-expressed in this cancer. The ceRNA network was constructed for normal and tumor states respectively of each cancer type.

### Functional analysis

The functional enrichment analysis were used to understanding the functional roles of lncRNAs in the ceRNA network. In this study, we used the cumulative hypergeometric test to evaluate the significance of lncRNAs competitively regulated mRNAs that enriched pathways/cancer hallmark GO terms. The cumulative hypergeometric test formula can be represented as follow:

$$p=1-\sum_{k=0}^{m}\frac{\left(\begin{array}{c} n\\ k \end{array}\right)\left(\begin{array}{c} N-n\\ M-k \end{array}\right)}{\left(\begin{array}{c} N\\ M \end{array}\right)}$$ (3)

Where *N* is all of the genome-wide genes, *M* is the number of a given pathway/GO term genes that annotated in the *N* genes, *n* is the number of competing protein coding genes of a particular lncRNA for cancer hallmark GO term enrichment analysis and n represents the number of all protein-coding genes that participate in cancer specific ceRNA interactions for KEGG pathway enrichment analysis, *m* is the number of the competing protein coding genes of a particular lncRNA or all protein-coding genes that participate in cancer specific ceRNA interactions annotated for the given cancer hallmark GO term/KEGG pathway.

The KEGG pathway enrichment analysis were performed to explore the function of cancer specific ceRNAs, which was carried out by our previously developed subpathwayMiner package [65]. Significance P-values for functional analysis were corrected by Benjamini-Hochberg method.

### Identification of ceRNA network modules

For the ceRNA network of each cancer, we identified biclique modules which consist lncRNAs and their competitively regulated mRNAs. A biclique module is a complete bipartite graph in which edges represent relationships between every vertex of one lncRNA set to every vertex of one mRNA set. The biclique module were identified by using the algorithm downloaded from the website of the Computational Biology Laboratory in the Department of Computer Science, Iowa State University (http://genome.cs.iastate.edu/supertree/download/biclique/).

### Risk score model

A univariate Cox regression analysis was performed to evaluate the association between the expression level of each lncRNA/PCG in module of ceRNA network and the survival. Then, we used a risk score model to evaluate the association between survival and the combination of lncRNAs and PCGs in network model. The risk score for a network module was calculated as follows:

$$\text{Risk score}=\sum_{i=1}^{n}r_{i}\mathrm{Exp}\left(i\right)$$ (4)

where r_*i*_ is the Cox regression coefficient of nodes (lncRNA/PCG) *i* in the network module, *n* is the number of nodes (lncRNAs/PCGs) in the network module, Exp(*i*) is the expression value of node i in the corresponding samples. The median value of risk score was used as cut-off to classify patients into high and low-risk groups.

### Survival analysis

We performed survival analysis on the ceRNA modules for each cancer. First, K-mean clustering method was used to classify the tumor samples of each cancer type into two groups based on the expression of the genes and lncRNAs in each module. Then, Kaplan-Meier estimate method was used to evaluate the survival difference of the two groups, and the significance was estimated by the log-rank test.

## SUPPLEMENTARY FIGURES AND TABLES


