# Supplementary material for: Comprehensive characterization of lncRNA-mRNA related ceRNA network across 12 major cancers
Source: Oncotarget. 2016 Aug 26;7(39):64148–67. doi: 10.18632/oncotarget.11637 (PMC5325432; doi:10.18632/oncotarget.11637)
Supplement: Supplementary file 1 [file oncotarget-07-64148-s001.pdf]

Comprehensive characterization of lncRNA-mRNA related ceRNA network across 12 major cancers

SUPPLEMENTARY FIGURES AND TABLES

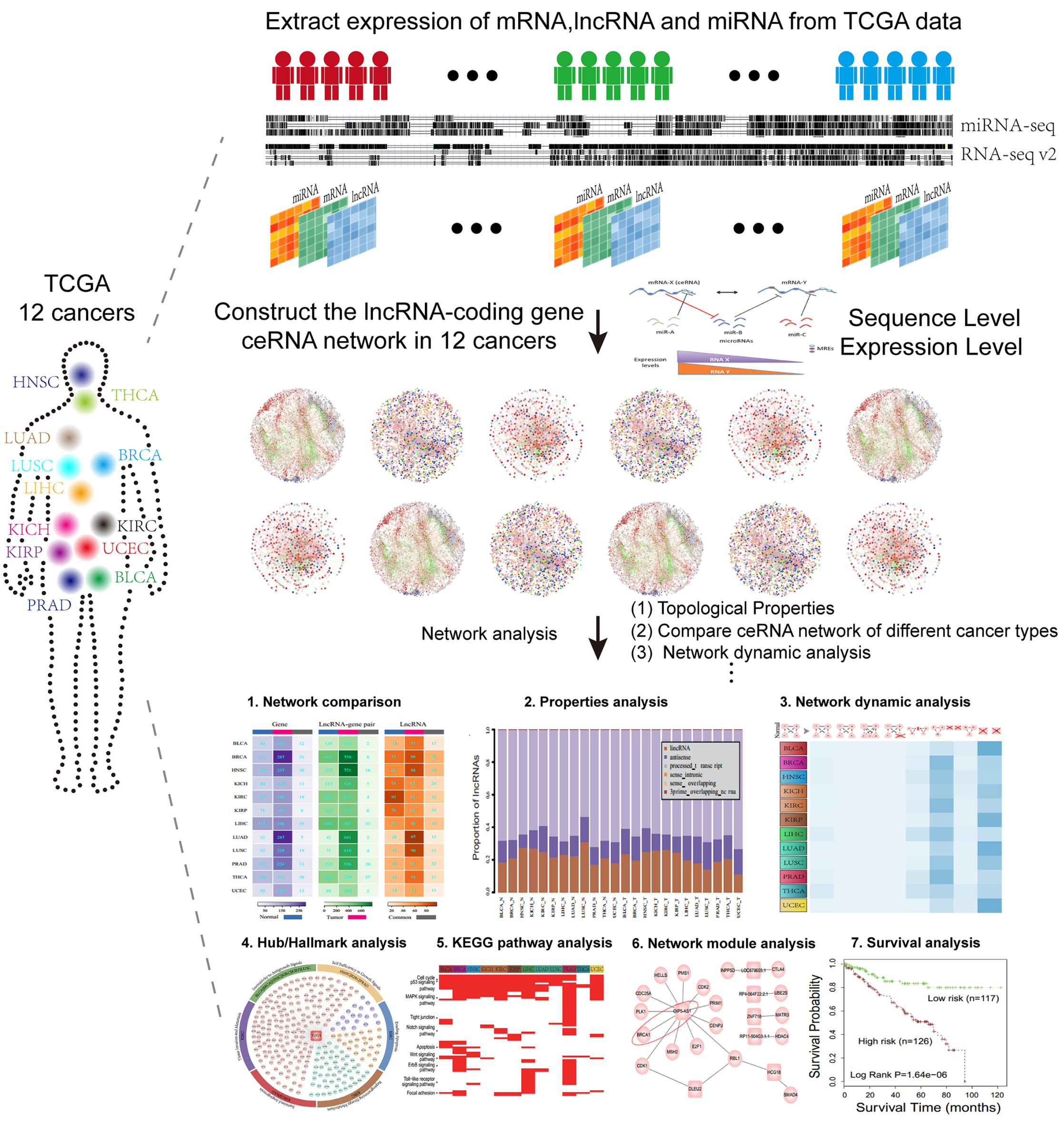

Supplementary Figure S1: The pipeline for constructing and analyzing the lncRNA related ceRNA networks in each cancer and normal state. (i) The lncRNA, miRNA and mRNA expression data of more than 5000 samples were extracted from TCGA. (ii) Integrated the interactome data and expression level data to constructed lncRNA related ceRNA networks in pan-cancer. (iii) Comprehensively characterized and analyzed the pan-cancer ceRNA crosstalk.

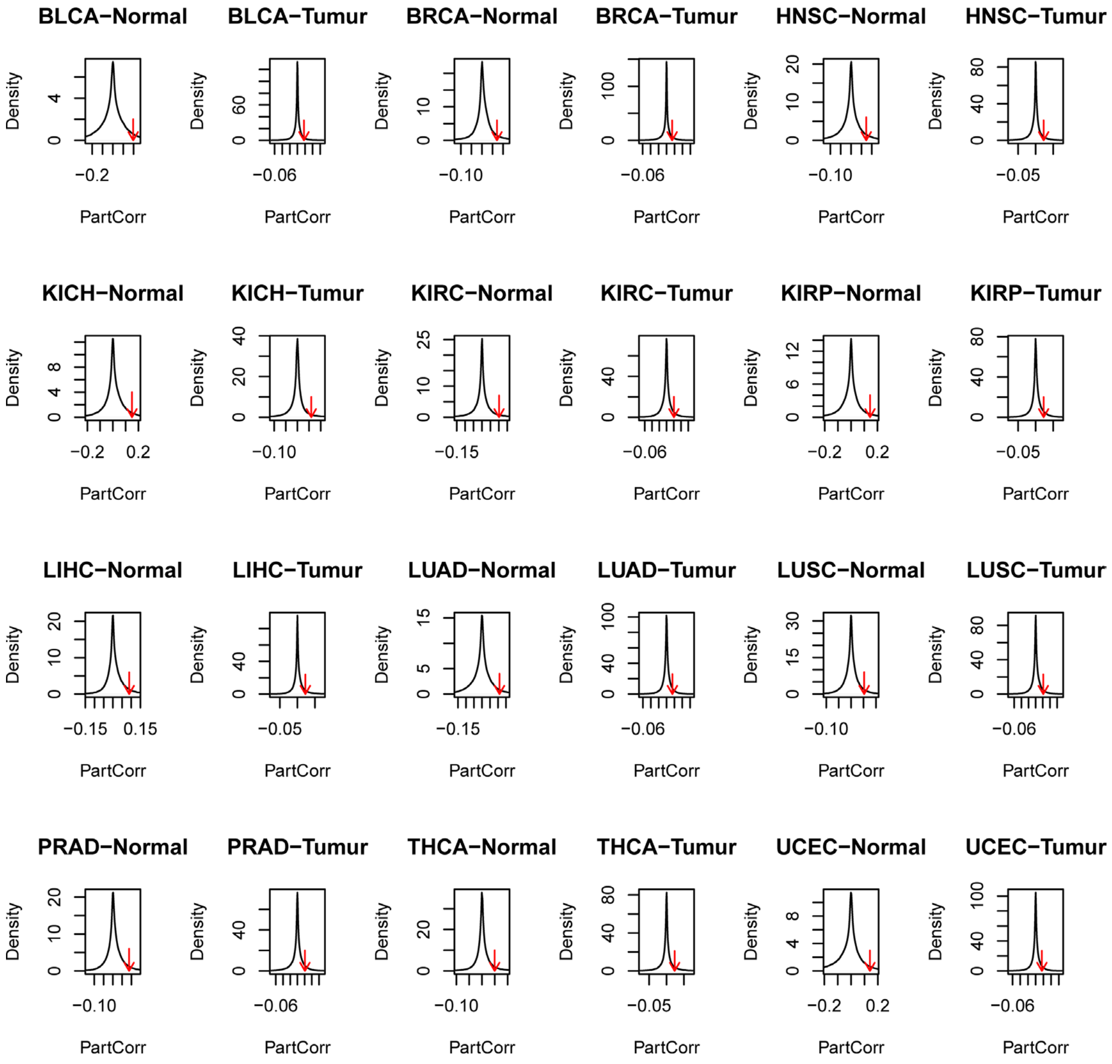

Supplementary Figure S2: The distribution of sensitivity score for each cancer types. The top 5% cut-off value of the random distribution were marked by red arrow.

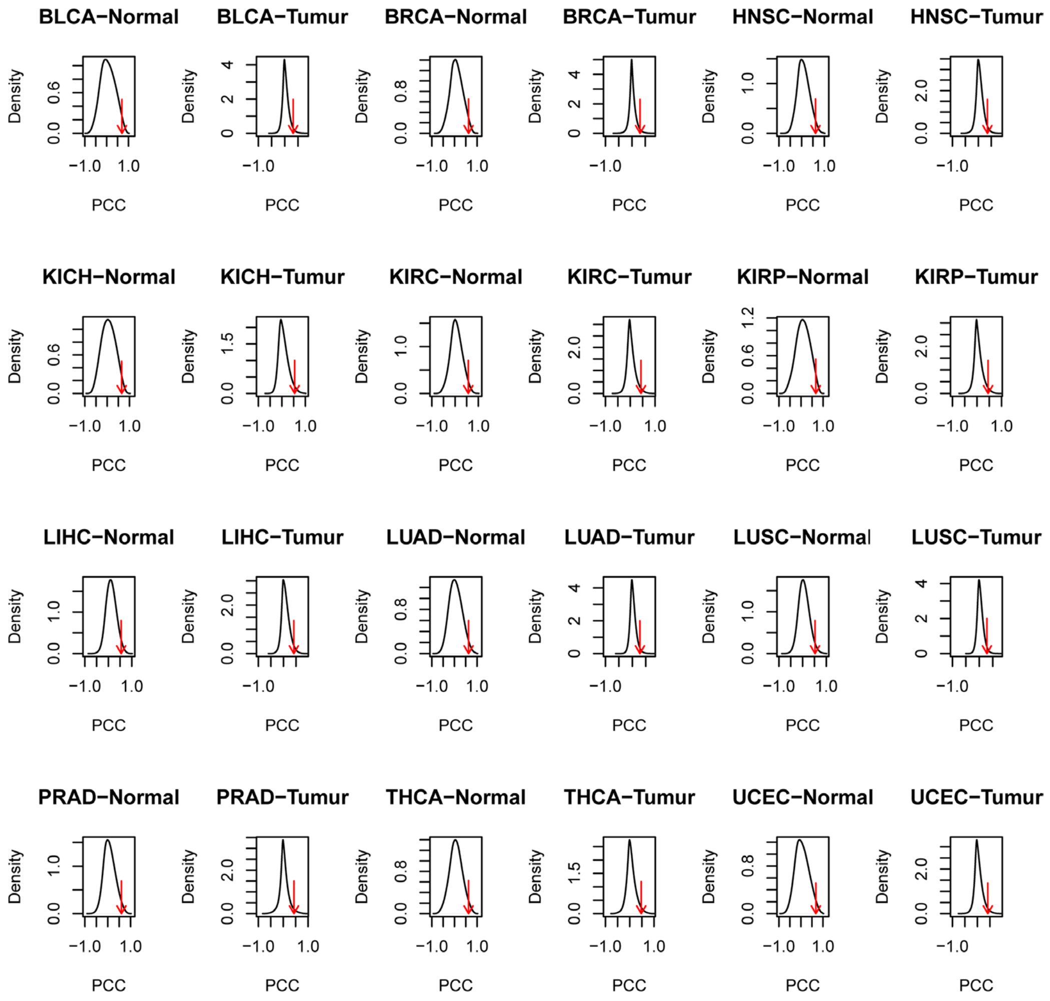

Supplementary Figure S3: The distribution of Pearson correlation coefficient of lncRNA-mRNA for each cancer types. The top 5% cut-off value of positive correlation were marked by red arrow.

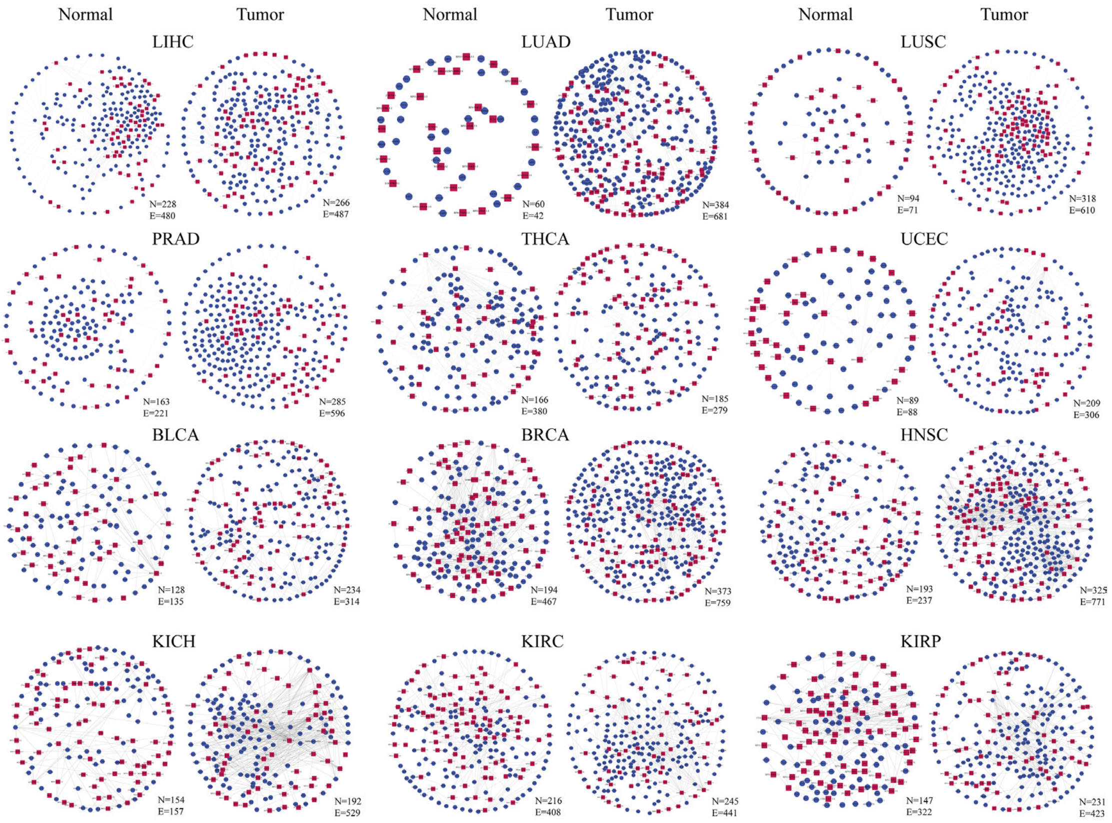

Supplementary Figure S4: Global landscape of lncRNA related ceRNA networks across 12 cancers. Blue and circle node represent PCGs, while red andrectangle nodes represent lncRNAs.

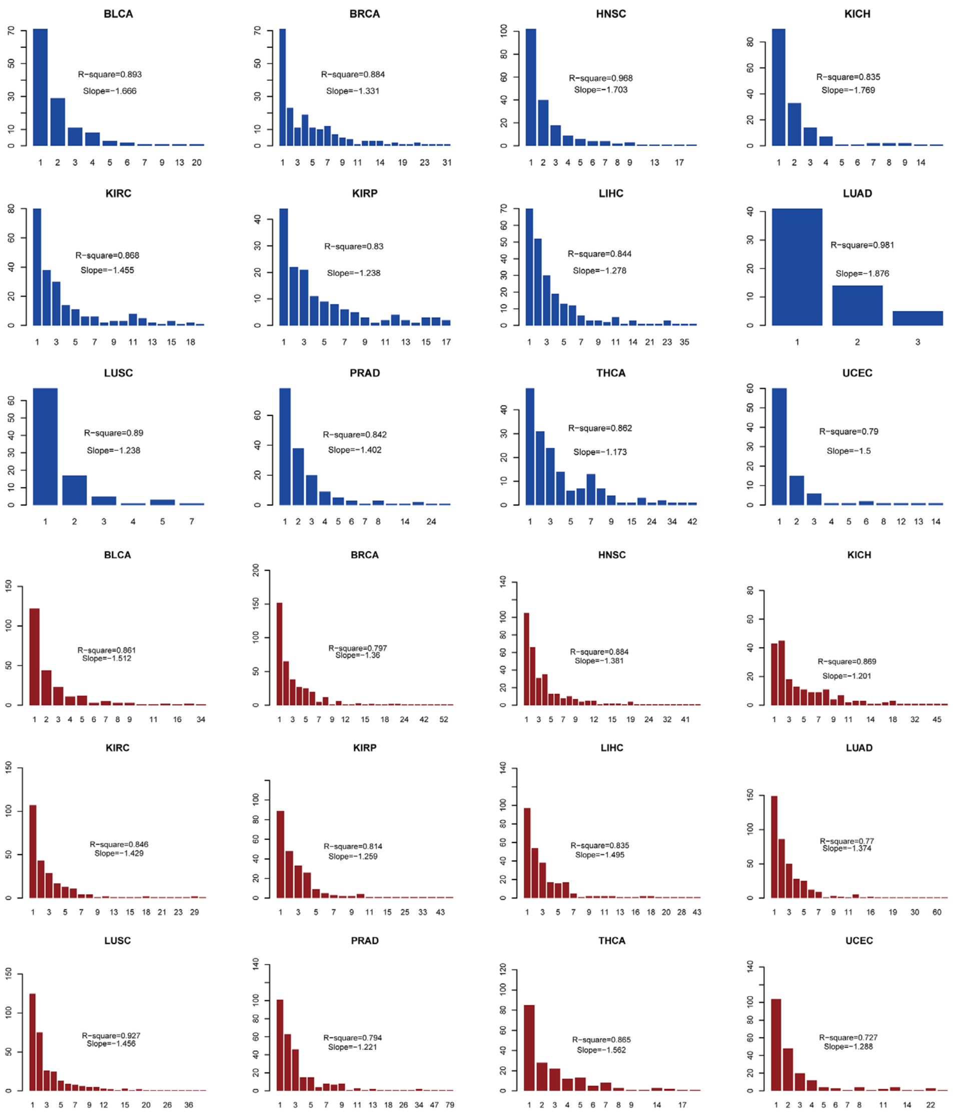

Supplementary Figure S5: Degree distribution of ceRNA netwokrs. Blue histograms represent degree distribution of Normal ceRNA netwokrs, while red histograms represent degree distribution of tumor ceRNA netwokrs. The slop (gamma) values of each ceRNA network were shown

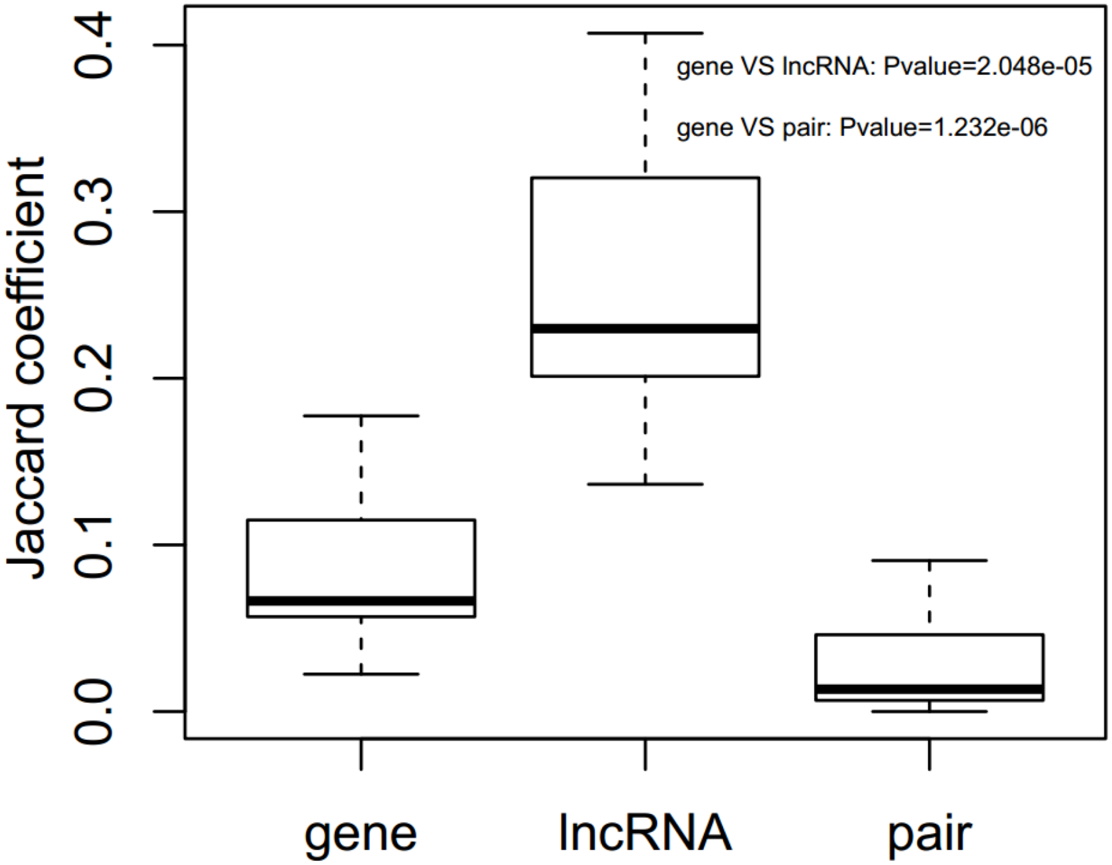

Supplementary Figure S6: Jaccard coefficient for each cancer type to measure the similarity between normal and tumor ceRNA network at the lncRNA, PCG (gene) and ceRNA pair levels respectively. Significance P-values were calculated by using T test.

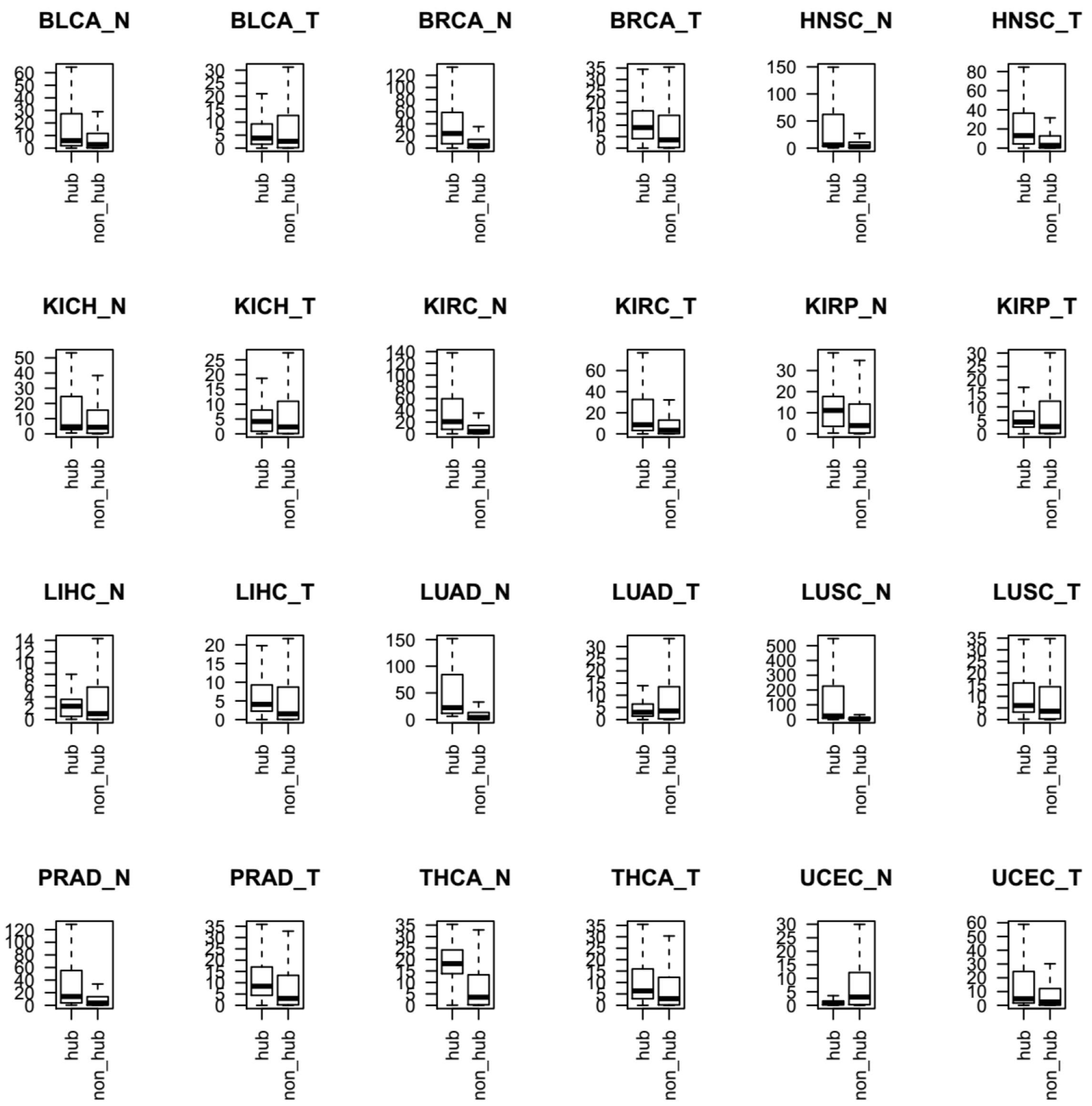

Supplementary Figure S7: The expression of hub PCGs across pan-cancer ceRNA networks are higher than non-hubs (other PCGs in network). The'_N' suffix represent normal ceRNA network, while '_T' suffix represent tumor ceRNA network.

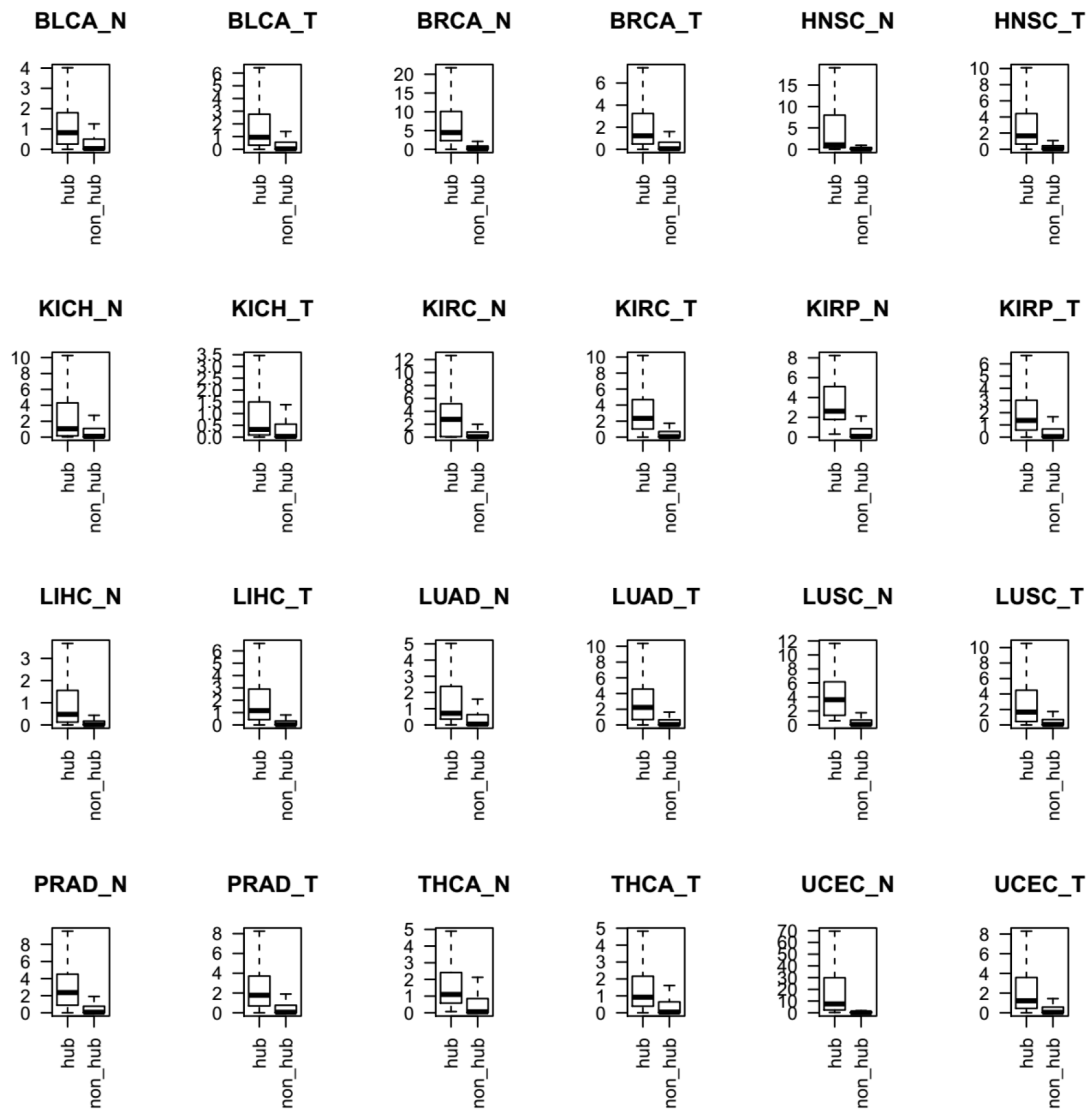

Supplementary Figure S8: The expression of hub lncRNAs across pan-cancer ceRNA networks are higher than non-hubs (other lncRNAs in network). The'_N' suffix represent normal ceRNA network, while '_T' suffix represent tumor ceRNA network.

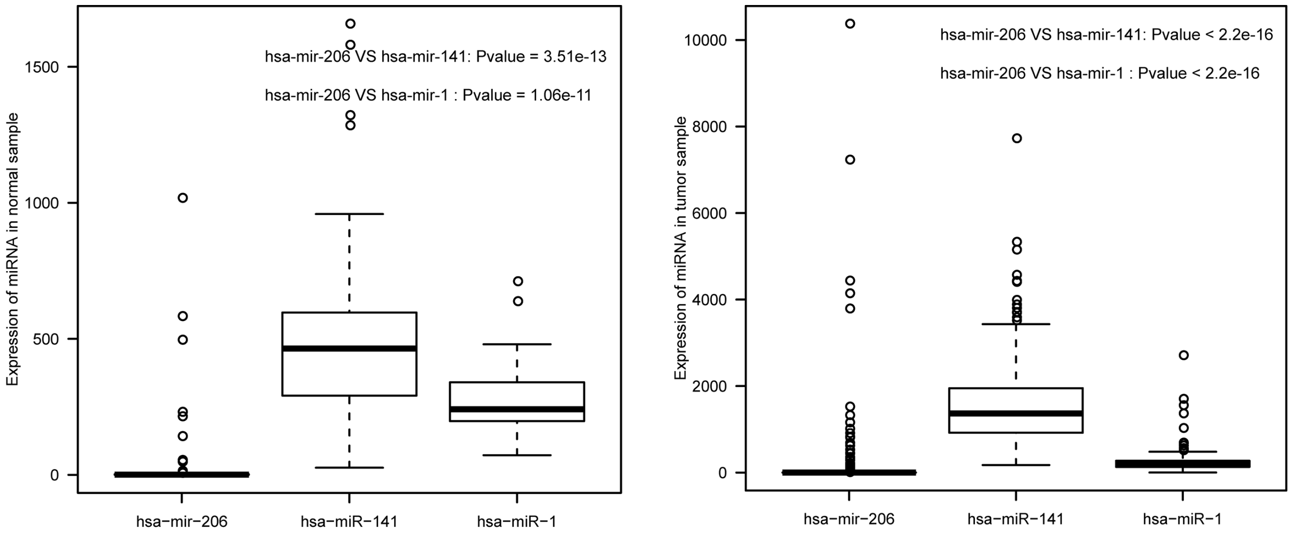

Supplementary Figure S9: Expression level of miR-206, miR-141 and miR-1 in tumor and normal states of PRAD. Significance P values were determined by the Mann-Whitney U test.

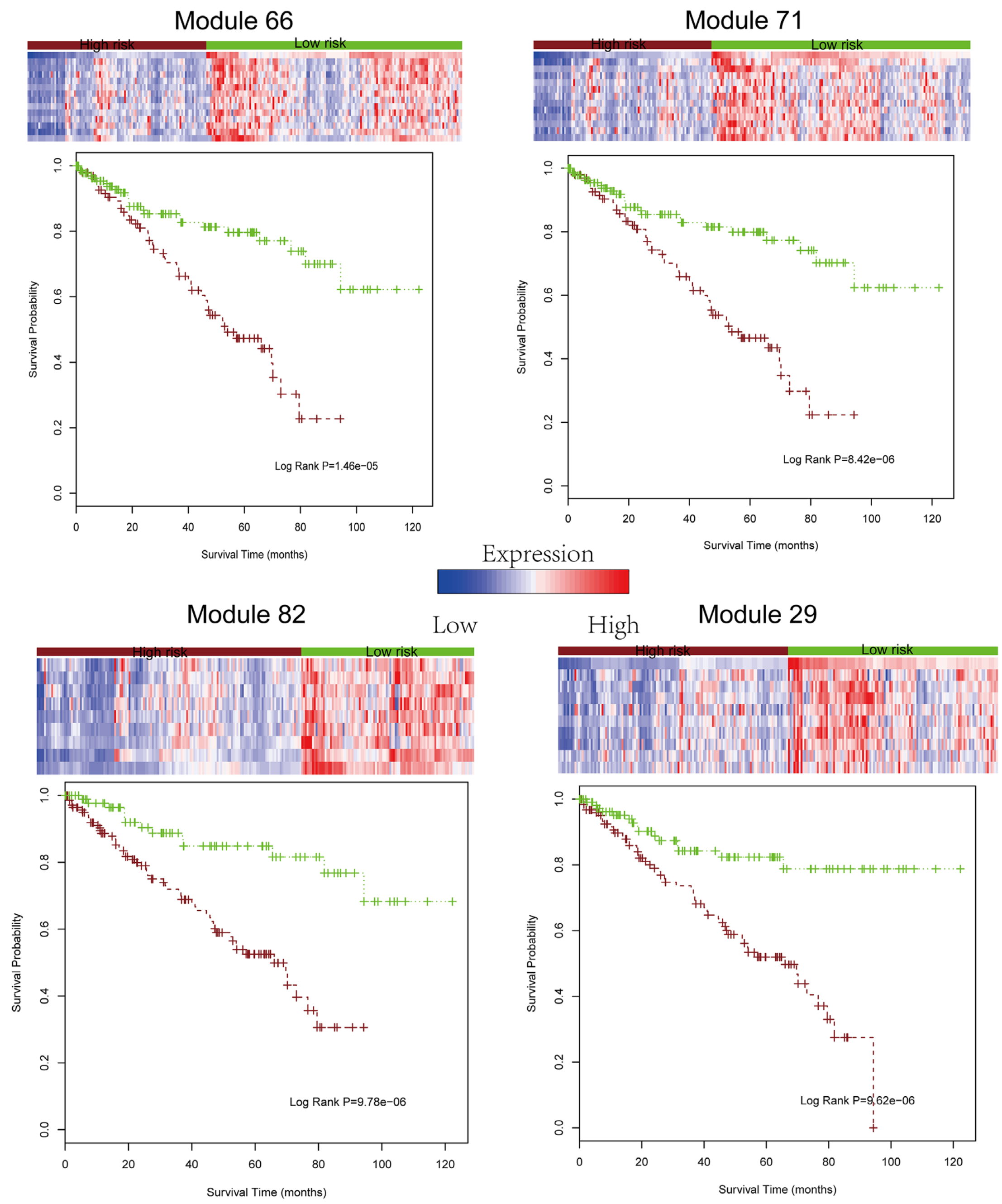

Supplementary Figure S10: Survival analysis for four ceRNA modules of KIRC. The significance of clinical outcome difference between the low-risk and high-risk groups was estimated by K-M survival analysis. P-values were calculated by the log-rank test.

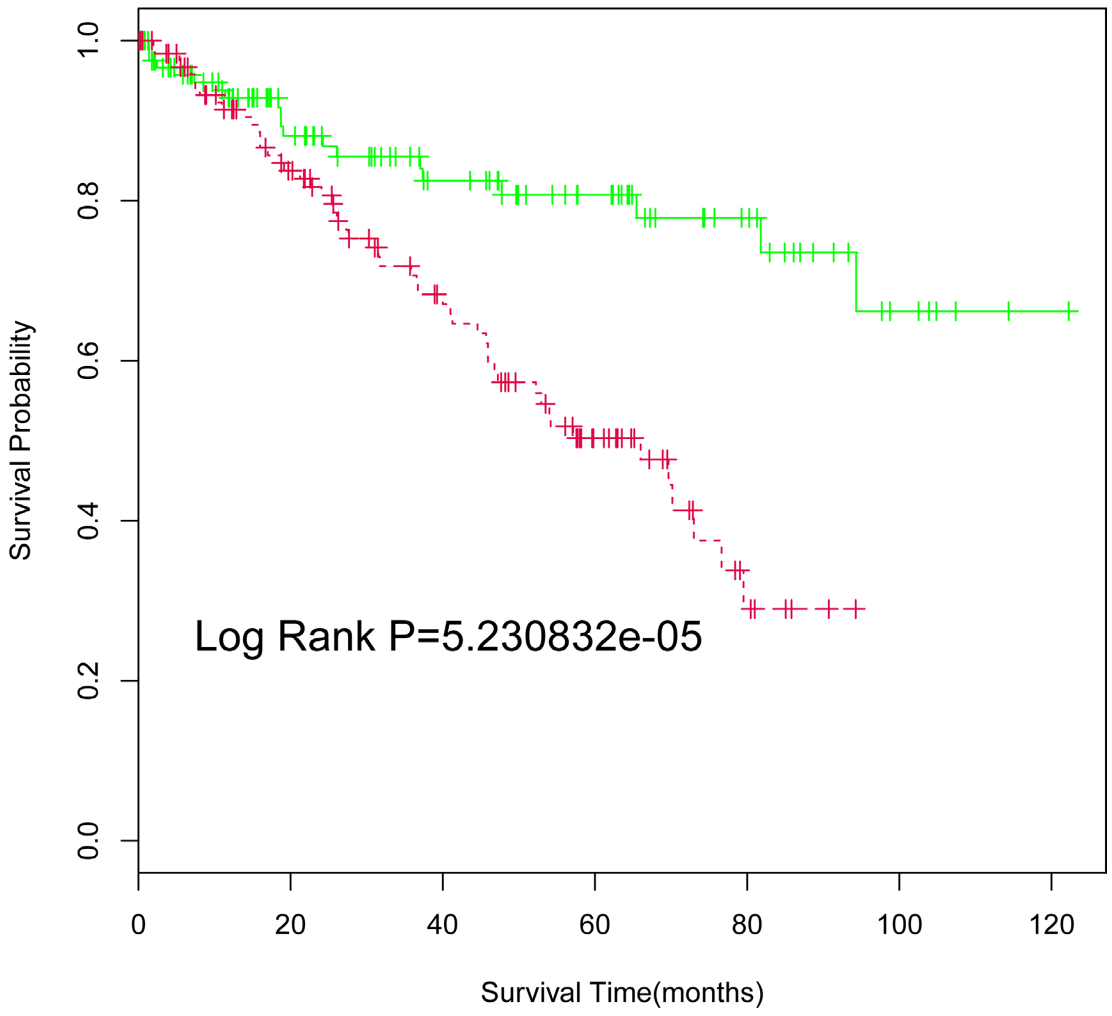

Supplementary Figure S11: Kaplan-Meier survival analysis of two groups of patients that reclassified by using the median value of risk score with different clinical outcomes.

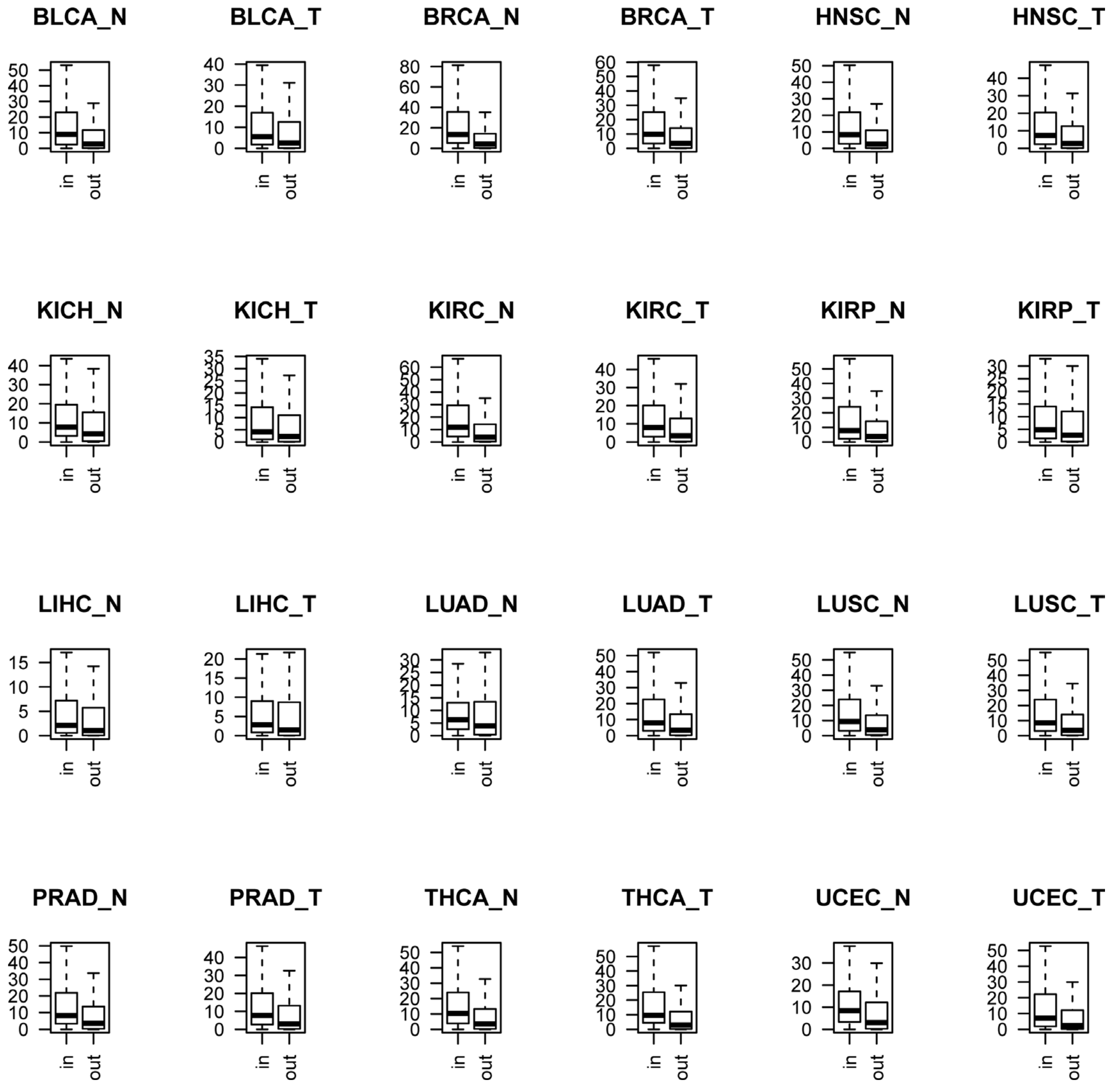

Supplementary Figure S12: The expression of PCGs in pan-cancer ceRNA networks are higher than other PCGs. The'_N' suffix represent normal ceRNA network, while '_T' suffix represent tumor ceRNA network.

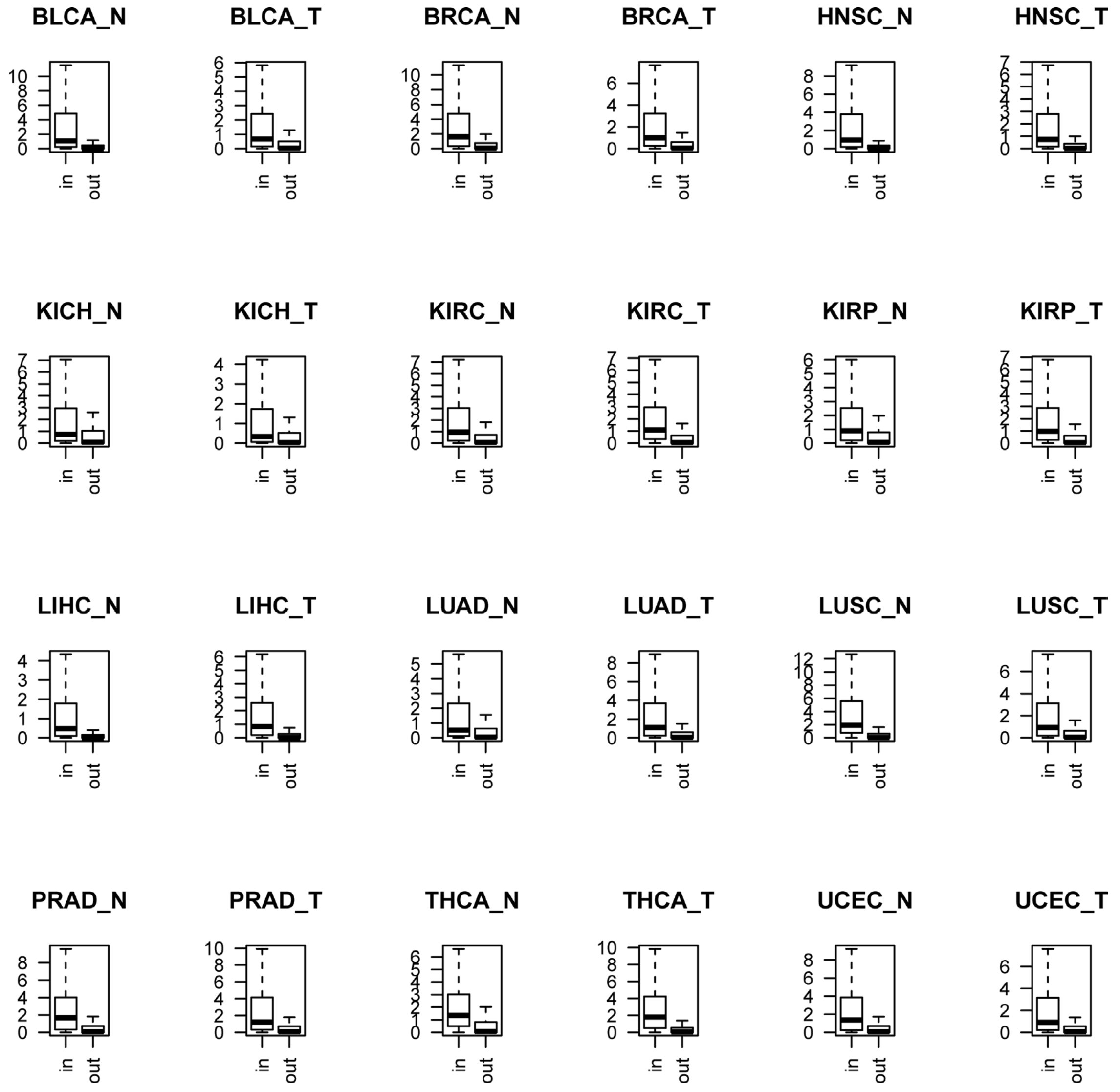

Supplementary Figure S13: The expression of lncRNAs in pan-cancer ceRNA networks are higher than other lncRNAs. The'_N' suffix represent normal ceRNA network, while '_T' suffix represent tumor ceRNA network.

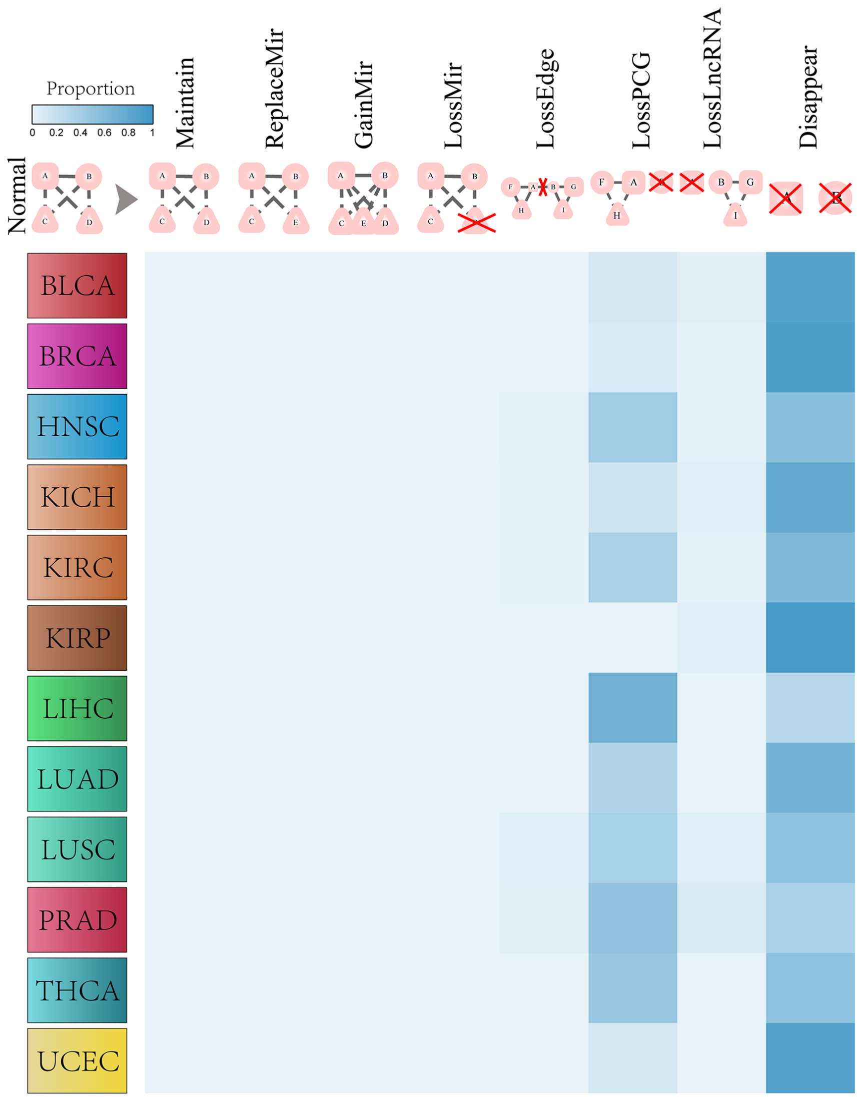

Supplementary Figure S14: The dynamic ceRNA interactions in normal and tumor states of pan-cancer. The ceRNA networks were constructed based on normal-matched samples for each cancer type.

Supplementary Table S1: Detailed sample information of 12 cancers

Tumor Type	Tumor Full Name	Tumor Samples	Normal Samples	Normal-matched samples
BLCA	Bladder Urothelial Carcinoma	405	19	19
BRCA	Breast invasive carcinoma	758	87	86
KIRP	Kidney renal papillary cell carcinoma	291	32	32
LUAD	Lung adenocarcinoma	445	19	12
LUSC	Lung squamous cell carcinoma	342	38	38
UCEC	Uterine Corpus Endometrioid Carcinoma	172	23	7
HNSC	Head and Neck squamous cell carcinoma	474	43	41
KICH	Kidney Chromophobe	66	25	25
KIRC	Kidney renal clear cell carcinoma	255	71	68
LIHC	Liver hepatocellular carcinoma	350	50	49
PRAD	Prostate adenocarcinoma	494	52	52
THCA	Thyroid carcinoma	463	53	51

Supplementary Table S2: The minimum threshold value of S that top 5% correspond to for each ceRNA networks

Cancer Type	S (Normal)	S (Tumor)
BLCA	0.20	0.02
BRCA	0.07	0.02
HNSC	0.07	0.022
KICH	0.15	0.06
KIRC	0.10	0.02
KIRP	0.14	0.02
LIHC	0.09	0.02
LUAD	0.11	0.015
LUSC	0.05	0.02
PRAD	0.09	0.02
THCA	0.05	0.02
UCEC	0.14	0.02

Supplementary Table S3: The minimum Pearson correlation coefficient, maximum P-value and FDR for significant ceRNA pairs in each ceRNA network

Cancer Type	Normal			Tumor		
	PCC	P-value	FDR	PCC	P-value	FDR
BLCA	0.70	0.00049	0.015	0.32	4.60E-11	1.50E-09
BRCA	0.62	2.09E-11	6.40E-10	0.30	6.08E-18	1.83E-16
HNSC	0.61	6.71E-06	0.00022	0.35	1.99E-15	5.68E-14
KICH	0.65	0.000278	0.0085	0.54	1.41E-06	4.78E-05
KIRC	0.57	1.15E-07	3.38E-06	0.43	2.46E-13	8.58E-12
KIRP	0.67	1.34E-05	0.00039	0.45	1.72E-16	5.58E-15
LIHC	0.55	2.55E-05	0.00071	0.41	2.15E-16	6.22E-15
LUAD	0.63	0.0033	0.11	0.28	1.18E-09	3.93E-08
LUSC	0.53	0.00055	0.017	0.28	8.44E-08	2.72E-06
PRAD	0.60	1.29E-06	4.10E-05	0.43	8.86E-25	2.39E-23
THCA	0.62	2.48E-07	7.06E-06	0.47	2.98E-28	8.80E-27
UCEC	0.68	0.00022	0.0074	0.42	8.33E-09	2.73E-07

Note: PCC: minimum Pearson correlation coefficient; P-value: maximum Fisher's asymptotic p-value; FDR: maximum corrected P-value.

Supplementary Table S4: Comparison of ceRNA network between tumor and normal states of each cancer type

Cancer Type	Pair normal	Pair tumor	Pair intersect	Triplets intersect
BLCA	135	314	2	2
BRCA	467	759	8	3
HNSC	237	771	18	10
KICH	157	529	5	5
KIRC	408	441	4	3
KIRP	322	423	0	0
LIHC	480	487	32	31
LUAD	42	681	2	1
LUSC	71	610	4	3
PRAD	221	596	20	11
THCA	380	279	27	26
UCEC	88	306	2	2

Note: Pair normal (tumor) represents the number of lncRNA-mRNA ceRNA pairs in each network; Pair (Triplets) intersect represents the number of shared lncRNA-mRNA pairs (lncRNA-miRNA-mRNA triplets) between normal and tumor network for each cancer type.

Supplementary Table S5: Jaccard coefficient of competing triplets between tumors with similar tissue origin

Cancer pair	Pair Jcard	Triplets intersect	Triplets Jcard
KIRC-KIRP_Normal	0.11	73	0.11
KIRC-KICH_Normal	0.06	32	0.06
KIRP-KICH_Normal	0.04	17	0.04
LUAD-LUSC_Tumor	0.08	103	0.07

Note: 'Pair (Triplets) Jcard' represents the Jaccard coefficient of lncRNA-mRNA pairs (lncRNA-miRNA-mRNA triplets) for the two corresponding netwroks. 'Triplets intersect' represents the shared number of lncRNA-miRNA-mRNA triplets for the two corresponding netwroks.

Supplementary Table S6: The minimal degree value of hub nodes for each ceRNA network

	Minimal degree of hub nodes (normal)	Minimal degree of hub nodes (tumor)
BLCA	4	7
BRCA	12	10
HNSC	6	12
KICH	4	14
KIRC	11	8
KIRP	12	8
LIHC	9	7
LUAD	3	7
LUSC	3	11
PRAD	6	9
THCA	8	7
UCEC	4	7

Supplementary Table S7: Comparison of ceRNA networks constructed based on normal-matched samples

Cancer Type	PCG			LncRNA-PCG pair			LncRNA		
	Normal	Tumor	common	Normal	Turmor	common	Normal	Tumor	common
BLCA	23	21	1	27	65	0	11	32	3
BRCA	14	76	1	17	108	0	6	49	4
HNSC	24	65	2	35	110	0	23	35	11
KICH	21	81	5	23	181	0	16	40	8
KIRC	31	119	2	40	363	0	19	28	5
KIRP	14	53	2	16	71	0	10	23	0
LIHC	53	43	0	73	65	0	22	21	11
LUAD	23	14	0	27	16	0	15	11	2
LUSC	59	26	2	123	40	0	47	25	11
PRAD	37	33	2	60	54	0	24	27	10
THCA	13	40	0	25	73	0	11	18	4
UCEC	10	8	0	11	9	0	5	8	1
